# Gender norms in sexual and reproductive health and rights: insights from young Angolan women and the development of a context-specific questionnaire (2021–2022)

**DOI:** 10.1186/s13690-025-01820-z

**Published:** 2025-12-23

**Authors:** Gunilla Priebe, Arciolanda Macama, Francisca Van Dunem dos Reis, Maria Malomalo, Jeanette Melin, Barbora Kessel

**Affiliations:** 1 School of Public Health and Community Medicine, University of Gothenburg, Gothenburg, Sweden; 2 Faculty of Economics and Management, The Catholic University of Angola, Luanda, Angola; 3 National School of Public Health, Nova University of Lisbon, Lisbon, Portugal; 4 Associação Mwana Pwo, Saurimo, Angola; 5 Department of Health and Caring Sciences, Linnaeus University, Kalmar, Sweden; 6 Department of Leadership, Demand and Control, Swedish Defence University, Solna, Sweden

**Keywords:** Angola, Cross-sectional study, Gender norms, Intersectionality, Questionnaire, Young women

## Abstract

**Background:**

Restrictive gender norms and persistent inequalities continue to constrain women’s lives globally, particularly in relation to sexual and reproductive health and rights (SRHR). In Angola, despite international and national commitments, young women face enduring SRHR challenges, including high rates of adolescent pregnancy and limited access to contraception and maternal healthcare. These reflect systemic health constraints and the normative structures shaping expectations, behaviours, and entitlements. Addressing SRHR thus requires attention to both service provision and the gendered norms that sustain inequality. Yet, measuring gender norms remains methodologically complex: many instruments conflate individual attitudes with collective expectations or rely on indirect proxies, reducing conceptual precision.

**Methods:**

To address this gap, a mixed-methods study incorporating Rash analysis was undertaken. Drawing on a literature review and qualitative approaches, contextually salient SRHR topics were identified, and an eleven-item gender norms questionnaire was developed. The questionnaire was embedded in a larger cross-sectional survey of 2,081 young women aged 18–24 in urban and rural areas across three provinces. Descriptive statistics were applied to socio-demographic variables and gender norm items.

**Results:**

Provincial contrasts emerged: participants in better-resourced provinces reported lower rates of adolescent pregnancy and intimate partner violence, alongside stronger normative support for SRHR. Endorsement was most consistent for education and bodily autonomy, while norms relating to reproductive rights and equality within relationships were more contested. Rasch analysis indicated sound psychometric properties and adequate coverage of the latent construct, although some provincial variation in item functioning was observed.

**Conclusions:**

Psychometric techniques identified minor tensions within and between SRHR topics; yet overall, the questionnaire demonstrated suitability as a composite measure. Thematic and geographical variations in perceived normative support highlighted how gender norms intersect with broader inequalities. Integrating the socio-demographic analysis, and an aggregate gender norms index, and item-level differentiation provides a strong basis for intervention design. Such integration can support contextually grounded, gender-empowering initiatives that strengthen young women’s SRHR within their specific socio-economic realities.

**Supplementary Information:**

The online version contains supplementary material available at 10.1186/s13690-025-01820-z.

**Table:** 

Text box 1. Contributions to literature
• This study illustrates how gender norms linked to sexual and reproductive health and rights (SRHR)—widely understood to shape everyday life—can be examined through careful empirical measurement.
• Demonstrates the value of participatory mixed-method design and modern psychometric techniques, a still rare approach that improves clarity and validity in assessing norms.
• Deepens understanding of SRHR related gender norms for young women in Angola, an under-researched setting with high gender inequality and under-resourced maternal healthcare.
• Offers insights into the presence of restrictive gender norms and underscores the importance of intersectional approaches that respond to the structural conditions in each locality.

## Background

### The interplay of gender norms and sexual and reproductive health and rights

Gender inequality, sustained by restrictive social norms and personal attitudes, remains widespread and continues to constrain women’s and girls’ opportunities [1–10]. Gender norms—understood as socially shared expectations of appropriate behaviour for women and men, institutionalised within structures and internalised through social interaction [10]—display notable global commonalities [11–16]. Masculinities are frequently associated with autonomy, authority, and economic provision, whereas femininities tend to be linked to caregiving, adornment, and fragility. Such essentialised representations reproduce asymmetries of power, responsibility, and recognition between genders [11–16]. They shape access to material and symbolic freedoms, influencing agency, health-related behaviour, and engagement with healthcare; consequently, they are increasingly recognised as critical determinants of sexual and reproductive health and rights (SRHR) outcomes [17–22].

SRHR encompasses physical, mental, and emotional well-being, situating identity and embodiment within broader social and institutional relations. Grounded in principles of bodily integrity, autonomy, and respect, SRHR affirms the right to safe, consensual relationships and to reproductive decision-making free from coercion or discrimination [1, 5, 9–11]. Findings from the World Values Survey further illustrate the persistence of individual attitudes consistent with restrictive norms, particularly in relation to SRHR: while 28% of respondents expressed discriminatory views regarding women’s education, an even higher proportion (58%) held prejudicial attitudes towards reproductive rights [9].

Public health and development frameworks consistently identify SRHR and gender equality as foundational to human well-being, sustainable development, and the realisation of human rights [1–7, 17, 18, 23–25]. In Angola, the contextual focus of this study, women’s movements have long situated SRHR within broader struggles against the enduring gendered effects of colonialism and socio-economic inequality [8, 26–30]. Human rights organisations similarly frame reproductive rights as both moral and legal imperatives, emphasising that “valuing human life begins precisely where life is generated” [30].

Yet significant challenges persist. Angola continues to record one of the world’s highest maternal mortality rates [31], reflecting structural constraints such as limited access to maternal healthcare [32–37], inadequate contraceptive availability and SRHR education [38–41], and a high prevalence of intimate partner violence (IPV) [41–44]. Pregnancies among girls under 14 years—an age group facing sharply elevated maternal and neonatal risks [45–50]—are reported more frequently in Angola than elsewhere [31].

While official data and independent studies vary in their assessment of progress, both highlight persistent socio-economic and regional inequalities in access to SRHR services [31–37]. These disparities mirror broader gender gaps in literacy, economic participation, and food security [30–32, 37, 40–44, 50–54]. Consequently, Angola ranks 28th of 36 sub-Saharan African countries in gender equality, with women estimated to have 36% less access to opportunities than men [54]. Central to these dynamics are social norms that both reflect and reproduce restrictive understandings of gender roles and entitlements [6–14, 26–30].

Addressing such interrelated challenges requires multidimensional strategies that move beyond biomedical and service-oriented paradigms, recognising how social values and expectations shape health behaviours and access to care [55–59]. Alongside direct health interventions, these approaches seek to transform the structural and normative conditions that constrain women’s well-being—challenging, for instance, the normalisation of child marriage or limitations on women’s decision-making [1–8, 23, 60–62]. This perspective underscores that barriers to SRHR are not only material or institutional, but also deeply embedded within the normative frameworks that structure everyday life and institutional practice [1–8, 23, 60–62].

### Advancing the measurement of gender norms within SRHR

Although the influence of gender norms on lived experience is well established, their empirical measurement remains limited [22, 56, 59, 62, 63]. The multidimensionality of SRHR complicates efforts to capture normative mechanisms with conceptual and contextual sensitivity. Notably, several indicators under SDG 5—addressing gender discrimination and harmful practices—still lack robust measures [20].

Existing approaches often conflate individual attitudes with collective norms, obscuring the social embeddedness of normative influence [10, 20, 22]. Others are confined to particular domains [12, 63, 64] or rely on indirect proxies, such as health disparities [24, 25, 60, 63, 65]. Many also lack theoretical coherence and contextual attunement, limiting understanding of how norms operate and persist [10, 20, 62]. Furthermore, modern psychometric techniques, including Item Response Theory and Rasch analysis, remain underused despite their potential to enhance conceptual precision and validity [20, 64, 66, 67].

Further empirical work is needed to explore how gender norms function across contexts and domains [24, 62]. A promising direction involves examining socially embedded expectations within specific spheres of life, with SRHR providing a particularly salient lens. Understanding how widely such norms are shared can illuminate their influence and durability [22]. Contextually grounded analysis can produce more nuanced accounts of normative dynamics, mitigate conceptual distortion, and clarify the structural conditions sustaining gendered experiences. Ultimately, such insights may inform the design of interventions that are both effective and contextually responsive [9, 23, 60, 68, 69].

### Aim and objectives

Despite broad acknowledgement of gendered inequalities, empirical research on SRHR-related gender norms remains limited. In Angola, the scarcity of reliable data on women’s social conditions has been identified as a barrier to national development [8]. This study seeks to contribute to addressing this gap through three interrelated objectives.

First, to deepen understanding of how gender norms are perceived to influence SRHR within the Angolan context. Second, to explore the extent to which young women perceive these norms to be present in their everyday environments, specifically the degree to which prevailing norms support or constrain their SRHR. Third, to identify methodological challenges and opportunities in developing a contextually grounded questionnaire for assessing gender norms within this complex and sensitive field.

Together, these objectives aim to strengthen conceptual and measurement approaches to gender norms and to inform future research and interventions promoting young women’s SRHR in Angola and similar settings.

## Methods

### Study setting

Angola, located on the southwest coast of Africa, is characterised by pronounced geographic, social, and economic diversity. Since independence from Portugal in 1975, the country has made significant progress in post-conflict reconstruction following a prolonged civil war that ended in 2002. Despite its substantial oil and diamond resources, development remains uneven, with nearly half of the population living in extreme poverty [70–72].

This study focuses on three provinces that exemplify these disparities [73–75]. Luanda, the capital province, is the country’s economic and political centre, characterised by rapid urbanisation and stark socio-economic divides. Huambo and Lunda Sul, both predominantly rural, face distinct yet interconnected challenges [32]. Huambo, with its strong agricultural base, continues to encounter barriers to poverty reduction and equitable service delivery [73]. Lunda Sul, noted for its diamond mining industry, reflects the paradox of resource wealth alongside limited infrastructure and persistent economic hardship [74, 75]. Across all the provinces, disparities in healthcare, education, and social services reveal enduring structural inequities [30–44, 70–75].

### Study design

This research forms part of the SADIMA project (an abbreviation of the Portuguese Saúde e Direitos das Mulheres em Angola), which examines the social determinants of young Angolan women’s SRHR, as well as their psychological well-being [75]. The present study employed a mixed-methods design, integrating qualitative approaches to explore gender norms, inform the development of a questionnaire, and implement it within a study involving young women in Angola. The identification of SRHR topics and the subsequent development of corresponding gender norm items proceeded through three main stages: (1) identification of SRHR topics and initial item development, (2) refinement and piloting of questionnaire items, and (3) assessment of measurement properties. The first two stages were undertaken alongside the broader questionnaire development for the SADIMA project. The designs and procedures specific to each phase are described under the corresponding subheading below.

### Identification of SRHR topics and initial item development

This phase combined a literature review with key informant interviews to identify priority SRHR topics. Through a participatory process oscillating between inductive and deductive reasoning, specific questionnaire items were developed, and the initial content validity of emerging constructs were assessed.

The process began with a review of relevant empirical, theoretical, and applied literature, which also informed the design of the overarching SADIMA survey instrument. This review highlighted the centrality of SRHR to women’s broader living conditions [1] and the persistent, unmet SRHR needs of young women in Angola [29, 34–38]. The focus on gender norms within the SADIMA project was further guided by priorities articulated by Angolan civil society organisations (CSOs) [8, 27, 30, 76, 77]. In addition, an examination of established gender-related scales [64] and key theoretical contributions on gender and intersectionality [14, 78–84] informed the conceptual development of the gender norms questionnaire.

To ensure contextual relevance, key informant interviews were conducted between July and September 2021 with representatives of governmental health institutions (*n* = 10), national CSOs (*n* = 8), and international governmental and non-governmental organisations (*n* = 7) operating in Angola (an additional file shows this in more detail [see Additional file 1]. A snowball sampling strategy was employed, initiated through contacts within national and provincial public health units. Interviews were conducted by a team of five researchers—four Angolan and one Swedish—with interdisciplinary expertise in public health, gender studies, and sociology.

A semi-structured interview guide, informed by [83–85], facilitated discussion on key challenges to women’s health and rights in Angola, ongoing initiatives, and perceived gaps in current efforts [see Additional file 1]. These insights were instrumental in assessing the extent to which SRHR topics identified in the literature were also prioritised—or not—by participants as critical issues affecting young women’s SRHR in the Angolan context.

### Refinement and piloting of questionnaire items

This phase involved the iterative development, refinement and piloting of the questionnaire’s gender norms items. Drawing on theoretical literature and participatory engagement, key SRHR topics were identified for inclusion.

Two participatory workshops were conducted to assess the relevance of proposed SRHR topics to prevailing gender norms: one with young female university students (*n* = 8) and another with young female data collectors (*n* = 12). The workshops also addressed ethical considerations, the use of inclusive language, and the conceptual clarity of items for the intended respondents. Feedback informed the exclusion of topics perceived as unrepresentative of widely shared social expectations or unlikely to be meaningful or recognisable to the broader population of young women. Topics identified as reflecting commonly held beliefs about appropriate behaviours for girls and women were reformulated as injunctive norm statements, capturing what others would approve or disapprove of [10, 22]. The resulting questionnaire aimed to document participants’ perceptions of SRHR-related expectations within their everyday environments, with these perceptions treated as meaningful reflections of prevailing gender norms.

This approach was guided by two premises. First, young women were considered active social agents, capable of reflecting on and articulating normative expectations. Second, while individual perceptions may not fully capture such community-wide views, they still shape self-concept, decision-making, and behaviour. To help participants distinguish between personal attitudes and perceived normative expectations, a standardised introduction was developed, encouraging responses based on “the ideas of people in this neighbourhood, village, or community,” a phrasing reiterated before each item to reinforce the social norms focus. An additional file shows this in more detail [see Additional file 2].

Revised items were subsequently field-tested within the broader SADIMA survey. Structured feedback was collected from interviewers and respondents following each interview (*n* = 66) and reviewed by two researchers. Daily debriefing sessions facilitated reflection on field experiences, identification of challenges, and refinement of data collection procedures. The piloting phase continued for two weeks, concluding when interviewers confirmed that all items and response options were clearly understood by participants. This process also assessed whether statements should be framed positively or negatively to reflect support for or opposition to SRHR principles and ensured comprehension of the Likert scale. Minor linguistic adjustments were made to enhance clarity; no new items were added, and none of the existing items were considered redundant or irrelevant [see Additional file 2].

### Assessment of measurement properties

In the third, and final phase, measurement properties of the proposed gender norms questionnaire were evaluated using a polytomous Rasch model (the partial credit model), drawing on data from the cross-sectional component of the SADIMA project.

#### Sampling and data collection

Details on the SADIMA study design, including sampling procedure, sample size, and data collection protocols, are provided in Priebe et al. [75]. This cross-sectional study included women aged 18–24. Sampling was informed by the latest DHS survey [32], and aimed to capture at least 1,885 young women across socio-economically diverse rural and urban settings in Luanda, Huambo and Lunda Sul [32].

Data were collected between February and May 2022 by trained female interviewers with university-level education. Interviewers had undergone a project-specific, one-week training programme covering questionnaire content, interviewing techniques, and research ethics. Participants received study information prior to giving informed consent. Each interviewer conducted approximately four interviews per day. To support data quality, daily consistency checks were performed, and weekly reviews were undertaken to detect and mitigate potential interviewer bias [75].

In addition to responses to gender norm items, which were recorded using a five-point Likert scale (totally agree / agree / it depends, neither agree nor disagree / disagree / totally disagree), the analysis included background characteristics and general SRHR indicators. Thirty questions from the broader SADIMA survey were used to construct relevant variables. Several of these were adapted from the Angolan DHS [86], including literacy (yes/no), formal education (high/medium/low), work (yes, permanent / yes, temporary / no), household wealth tertiles (3d/2d/1st), fertile period knowledge (yes/no), modern family planning awareness (yes/no), and modern family planning use (yes/no). The latter two variables, based on DHS definitions of modern family planning methods, encompassed condoms, oral contraceptives, injections, and implants [86].

Additional variables were derived from established scales, such as household night hunger (no/yes) [87] and exposure to intimate partner violence (no/yes) [88]. Other variables were developed specifically for the SADIMA study, conceptually informed by the DHS framework. These included province of residence (Luanda / Huambo / Lunda Sul), area of residence (urban / rural), pregnancy history categorised by age at first pregnancy (no / yes ≥ 18 years / yes, < 18 years), menstrual autonomy (yes/no: defined here as the ability to continue daily activities outside the home during menstruation), and acquisition of information about pregnancy and childbirth complications (yes/no). [see Additional file 2].

#### Psychometric analysis

The polytomous Rasch model describes the probability of selecting a given response option as a function of respondent’s position on the latent continuum of SRHR-supportive gender norms and the relative difficulty of endorsing each item, represented by item-specific threshold parameters. In this way, the internal structure of the items can be mapped onto respondents’ positions on the latent scale [66]. This allows for an assessment of whether the selected items are appropriate for measuring the construct within the target population, and it illuminates both the structure of the items and their relationships.

The polytomous Rasch model was fitted to data from participants who responded to at least three quarters (≥ 9) of the gender norms statements, providing sufficient information on their perception of gender norms. Following Johansson et al. [89], the analysis evaluated unidimensionality (including local response dependence, and item fit), ordering of response categories, measurement invariance, targeting, and reliability of the proposed questionnaire.

Unidimensionality was assessed through principal component analysis of the covariance matrix of unconditional standardized residuals, constructed elementwise using pairwise complete observations. Support for unidimensionality was inferred when the first eigenvalue was below 2 and the variability explained by subsequent components was low. Item fit was examined visually through conditional item characteristic curve (CICC) plots [90], grouping of participants into deciles based on total scores to compare expected and observed average scores for each item. This visual assessment was supplemented by outfit and infit statistics calculated from unconditional standardised residuals, estimated via subsampling (350 participants per sample, 100 repetitions) [91]. Item misfit was indicated when mean-square infit or outfit statistics fell outside 0.7–1.3, or normalized infit or outfit statistics exceeded ± 2.

Local dependence was investigated using Yen’s Q3 statistic, with item pairs considered dependent if the Q3 value exceeded the average by more than 0.2 [92]. The ordering of response categories, reflected in estimated threshold values, was evaluated through visual inspection of item characteristic curves. Categories were considered appropriately ordered when each response option was the most probable to be selected in sequence across the latent trait continuum [93].

Measurement invariance was examined with respect to province, household wealth, literacy, and area of residence. Differential item functioning (DIF) was explored using partial credit trees [94], with a stopping rule applied to avoid subgroup sizes smaller than 350. DIF magnitude was assessed visually using modified CICC plots, comparing expected scores under a common model with observed scores conditional on total score within identified subgroups. Threshold instability was visualised by plotting item thresholds across DIF subgroups. The impact of DIF on person location was evaluated by comparing estimates from a model, which kept as common only items functioning comparably across subgroups, with those from a common model ignoring DIF, with scale alignment achieved by fixing the first threshold of the first common item to zero. Agreement between person estimates was examined using Bland-Altman plots [95], and histograms visualised differences in trait distributions among DIF groups, with differences interpreted relative to the standard deviation of the location estimates.

Targeting was assessed by comparing the distribution of estimated person locations with item threshold distributions. Good targeting was considered present when item thresholds covered a similar range to that of person estimates on the latent scale. Reliability was evaluated using the test information function, with participants (with complete data) considered measured with satisfactory precision if test information exceeded 3.33, corresponding to a person separation index of 0.7 [89, 96].

All analyses were conducted using SPSS (version 29.0.0.0) and R (version 4.3.3) [97]. The psychometric analysis was conducted using *eRm* package (version 1.0–5) [98], *psychotree* package (version 0.16-0) [94], customized functions from *RASCHplot* (version 0.1.0) [99]. Visualisations were generated using *RISEkbmRasch* (version 0.1.34.1) [89] and a final figure processing used *cowplot* (version 1.1.3) [100] and *patchwork* (version 1.3.1) [101].

## Results

### Identification of SRHR topics and development of gender norm items

Table 1 presents the SRHR topics identified through qualitative analysis as particularly salient within the Angolan context. It also indicates which topics were judged sufficiently connected to prevailing gender norms—understood as socially embedded expectations—to justify their inclusion in the questionnaire. For each SRHR topic, the table outlines the associated human rights aspect, and the corresponding questionnaire item and specification. Topics excluded following participatory workshop discussions are listed in the lower section of the table.

**Table 1:** Sexual and reproductive health and rights topics identified through qualitative analysis of key informant interviews conducted in Angola in 2021, along with their corresponding items in the gender norms questionnaire, presented in survey order. Excluded items are listed separately

SRHR topics emphasised in the literature and key informant interviews	Item label	Questionnaire specification
	The right to…	Neighbourhood, village or community normative support for…
Education is a foundation for empowerment and informed decision-making, and supporting girls continued learning—by creating conditions that reduce their household labour and enable families to prioritise their education—is vital for advancing their health and rights.	education access	…girls’ education.
Women’s decision-making power within a relationship is a key factor in facilitating their meaningful participation in decisions that promote health and well-being, while also enhancing household planning and long-term family resilience.	decision equality*	…equal decision-making power between women and men in the household.*
Although sexual consent is not legally required within marriage, it remains central to the promotion of gender equality and the prevention of intimate partner violence.	marital consent	…a woman’s right to refuse sex with their partner for any reason.
Fostering supportive environments that enable women to express themselves freely and share diverse perspectives without fear of punishment or stigmatization is essential for advancing their rights, well-being, and meaningful participation in family and societal life.	freedom of expression*	…woman to question their partner’s opinion and still be respected.*
Childbearing is frequently valued as a source of joy, strength, and social significance for women; however, this emphasis may be experienced by some as pressure to have children, potentially impacting the self-worth of those who do not.	identity diversity*	…recognising that motherhood is not required for a woman to be considered a ‘real woman’.*
Acknowledging women who have experienced sexual abuse as entitled to support, dignity, and justice is crucial for creating a safe environment that encourages disclosure, facilitates help-seeking from trusted sources, and ensures access to the healthcare, counselling, and legal services necessary for healing and recovery.	non-stigmatisation*	…speaking about experiences of sexual abuse with family and friends without shame.*
Promoting girls’ right to a safe and healthy childhood—free from the adult responsibility of motherhood—alongside gender balanced responsibility for pregnancy prevention, is essential for supporting adolescent health, gender equality, and the realisation of their rights.	reproductive maturity*	…girls to delay childbearing until age 18.*
Acknowledging intimate partner violence as a health and rights issue—and ensuring it is treated as such by all sectors, including law enforcement—is essential for protecting the well-being of women and children, promoting justice, and strengthening coordinated responses and prevention efforts.	freedom from violence*	…women not having to tolerate partner violence to keep the family together.*
Upholding women’s rights to bodily autonomy, integrity and dignity—free from coercion or harassment—is fundamental to fostering safe environments across societal settings, where opportunities are equitably determined by merit rather than by deference to gendered authority.	bodily autonomy*	…women’s right to reject sexual demands from men in positions of power.*
Promoting shared responsibility for family planning and women’s reproductive health between women and their male partners is critical for achieving optimal pregnancy spacing and enhancing maternal and child health outcomes.	maternal health	…couples to limit pregnancies for the woman’s health.
Supporting girls’ autonomy to choose if, when, and whom to marry empowers them to make decisions that promote their health and rights, helping to reduce risks associated with early pregnancy, sexually transmitted infections, and intimate partner violence.	relationship autonomy	… women’s right to decide whom to marry or form a relationship with.

**SRHR topics excluded as a result of qualitative evaluation**	**Rationale**	
Strengthening health literacy in SRHR empowers individuals to make informed decisions and protects them from abuse and disease, promoting their overall well-being and autonomy.	sufficiently covered by ‘education access’	
Promoting more equitable sharing of caregiving responsibilities supports women’s access to healthcare, rest, economic opportunities, and self-care, thereby enhancing their overall health and well-being.	sufficiently covered by ‘decision equality’	
Shared responsibility in preventing unwanted pregnancies contributes to improved contraceptive uptake and more consistent use.	sufficiently covered by ‘decision equality’ and ‘maternal health’	
Men’s supportive involvement in women’s health during pregnancy and childbirth can help advance maternal healthcare participation, improve health outcomes, and contribute to healthier family life for all members.	sufficiently covered by ‘decision equality’ and ‘maternal health’	
Access to youth-friendly, adequately equipped health facilities that provide family planning and maternal care, plays a critical role in preventing disability, serious illness, and associated socio-economic impacts.	partly covered by ‘decision equality’; limitations in healthcare access viewed as a systemic rather than gender-specific issue	
Equally valuing girls and boys is fundamental to ensuring their future equal rights, opportunities, and protections.	not regarded as a local priority, as the number of children takes precedence over their gender	
The ability to self-define one’s gender identity and sexual orientation is vital to SRHR, given its ties to identity and the risk for significant stigmatisation when prevailing social norms are challenged.	limited definition clarity across study contexts (better suited to qualitative research)	

*Statements marked with an asterisk (*) carried a negative connotation in relation to women’s rights as presented in the survey. However, for the purposes of this table, the corresponding SRHR concepts are framed in a rights-promotive manner to enhance clarity. For analysis, the Likert scale for these items had the highest score at ‘totally disagree’, whereas the positively connotated items had the highest score at ‘totally agree’

The final selection comprised eleven SRHR topics deemed to reflect a clear and contextually meaningful connection to prevailing gender norms. The priorities emerging from the key informant interviews and workshops broadly corresponded with key topics in international scientific and human rights literature. The selected topics represented a relatively balanced mix of the two core SRHR domains: sexual and reproductive.

Health was primarily framed as an outcome of the realisation of rights. Accordingly, rights-related aspects were more explicitly articulated, while health-related aspects tended to be implicit and often intersected with wider rights-based concerns. Items in the sexual health and rights domain included relationship autonomy, bodily autonomy, and non-stigmatisation. Items within the reproductive health and rights domain encompassed identity diversity, reproductive maturity, and maternal health. Foundational and intersecting concerns—such as girls’ right to education and broader aspects of women’s status within intimate relationships, including freedom from violence, freedom of expression, marital consent, and equality in decision-making—were retained due to their perceived centrality to the realisation of SRHR in the local context.

Four topics were excluded due to conceptual overlap, perceived redundancy, or limited resonance across Angola’s diverse social and geographical settings. The issue of self-determination in relation to gender identity and sexual orientation, while vital to SRHR, was excluded because workshop participants indicated that it was unlikely to be widely recognised or conceptualised across all study areas. Another excluded topic concerned access to specific SRHR information and services, which was considered overly aspirational in a context where education and healthcare remain severely under-resourced. In this case, lack of access was regarded as reflecting systemic challenges rather than gender-specific barriers.

### Characteristics of study participants

Table 2 summarises the socio-demographic characteristics and selected SRHR indicators of the young women who participated in the study (*n* = 2,081 of 2,109 recruited; an additional file shows this in more detail [see Additional file 3]). The data highlighted marked disparities in living conditions across the three provinces. Overall, respondents in Luanda reported comparatively favourable conditions, whereas those in Lunda Sul experienced the greatest challenges, with Huambo positioned between these two contexts.

**Table 2:** Background characteristics related to socio-demographic and sexual and reproductive health and rights aspects among young women in Angola (2022), presented as percentages by province

Variable	Category	Luanda *n* = 910	Huambo *n* = 684	Lunda Sul *n* = 487	Total *n* = 2081
		%	%	%	%
Socio-demographic characteristics					
Literate	Yes	85.7	53.7	41.5	64.8
	No	13.8	44.9	58.3	34.5
	Missing	0.4	1.5	0.2	0.7
Formal education	High	61.0	32.0	19.5	41.8
	Middle	28.2	35.8	32.0	31.6
	Low	10.8	31.9	48.5	26.5
	Missing	0.0	0.3	0.0	0.1
Work	Yes, permanent	18.6	17.4	6.0	15.2
	Yes, temporary	31.8	29.4	15.0	27.1
	No	49.3	52.9	79.1	57.5
	Missing	0.3	0.3	0.0	0.2
Household night hunger	No	71.8	49.9	40.7	57.3
	Yes	28.0	49.4	59.1	42.3
	Missing	0.2	0.7	0.2	0.4
Household wealth tertile	3d (better off)	60.3	16.5	4.7	32.9
	2d	33.0	33.9	31.4	32.9
	1st	5.4	47.8	63.2	32.9
	Missing	1.0	1.8	0.6	1.3
Area of residency	Urban	94.0	44.3	26.3	61.8
	Rural	6.0	55.7	73.7	38.2
General SRHR characteristics					
Pregnancy	No	56.4	26.6	20.5	38.2
	Yes, 1st > 18	24.9	28.8	25.9	26.4
	Yes, 1st < 18	18.0	43.6	53.2	34.6
	Missing	0.7	1.0	0.4	0.7
Menstrual autonomy	Yes	61.9	69.6	27.5	56.4
	No	37.1	28.4	71.7	42.3
	Missing	1.0	2.0	0.8	1.3
Intimate partner violence	No	58.8	54.5	33.3	51.4
	Yes	37.1	43.0	66.1	45.8
	Missing	4.1	2.5	0.6	2.7
Fertile period knowledge	Yes	67.9	72.2	33.1	61.2
	No	31.1	26.5	66.1	37.8
	Missing	1.0	1.3	0.8	1.1
Acquired information about pregnancy or childbirth complications	Yes	66.9	67.8	29.4	58.4
	No	32.7	31.3	69.8	40.9
	Missing	0.3	0.9	0.8	0.6
Modern family planning awareness	Yes	97.7	89.0	72.3	88.9
	No	1.4	9.8	26.9	10.1
	Missing	0.9	1.2	0.8	1.0
Modern family planning use	Yes	55.7	39.0	30.2	44.3
	No	34.1	56.6	67.4	49.3
	Missing	10.2	4.4	2.5	6.5

For instance, literacy rates varied considerably: 85.7% of women in Luanda were literate, compared with 53.7% in Huambo and 41.5% in Lunda Sul. Similarly, household food security was reported to be highest in Luanda (71.8%) but substantially lower in Huambo (49.9%) and Lunda Sul (40.7%). These findings aligned with household wealth distribution, whereby the highest wealth tertile was predominantly composed of participants from Luanda, while large proportions of women from Huambo (47.8%) and Lunda Sul (63.2%) fell into the lowest tertile.

Challenges related to SRHR were evident in all provinces, though they appeared most acute in Lunda Sul. Early pregnancy was reported by 53.2% of respondents in Lunda Sul and 43.6% in Huambo, compared with 18% in Luanda. Experiences of IPV were also widespread, with prevalence highest in Lunda Sul (66.1%), followed by Huambo (43%) and Luanda (37.1%). Menstrual autonomy was reported by a majority of participants in Huambo (69.6%) and Luanda (61.9%), but only by 27.5% in Lunda Sul. Similarly, awareness of key reproductive health topics was relatively high in Luanda and Huambo. For example, 72.2% of respondents in Huambo and 67.9% in Luanda correctly identified the fertile period, compared with 33.1% in Lunda Sul. Awareness of modern family planning methods (condoms, oral contraceptives, or injectables) was widespread—97.7% in Luanda, 89% in Huambo, and 72.3% in Lunda Sul—but reported use was lower: 55.7% in Luanda, 39% in Huambo, and 30.2% in Lunda Sul.

### Prevalence of SRHR supportive gender norms

Table 3 presents the proportion of respondents reporting perceived community support for selected items which are listed in descending order according to the percentage of participants selecting 4 or 5 on the Likert scale, indicating normative support. Results are disaggregated by province.

**Table 3:** Perceived normative support for sexual and reproductive health and rights items, by province, expressed as the proportion of respondents who selected 4–5 on the Likert scale in the cross-sectional study with young women conducted in Angola in 2022

Item label	Questionnaire specification	Luanda	Huambo	Lunda Sul	Total
		*n* = 888	*n* = 646	*n* = 480	*n* = 2081
The right to…	Normative support for…	%	%	%	%
education access	…girls’ education.	74.3	82.3	70.4	76.0
relationship autonomy	…women’s right to decide whom to marry or form a relationship with.	78.7	59.9	66.9	69.8
bodily autonomy*	…women’s right to reject sexual demands from men in positions of power.*	73.3	69.3	54.4	67.6
freedom from violence*	…women not having to tolerate partner violence to keep the family together.*	67.3	54.4	48.3	58.6
non-stigmatisation*	…speaking about experiences of sexual abuse with family and friends without shame.*	51.4	58.2	51.7	53.7
identity diversity*	…recognising that motherhood is not required for a woman to be considered a ‘real woman’.*	61.9	47.7	31.6	50.1
reproductive maturity*	…girls to delay childbearing until age 18.*	61.0	46.8	23.8	47.6
maternal health	…couples to limit pregnancies for the woman’s health.	50.1	50.3	38.6	47.5
freedom of expression*	…woman to question their partner’s opinion and still be respected.*	49.9	47.1	37.6	46.1
marital consent	…a woman’s right to refuse sex with their partner for any reason.	44.3	37.4	48.9	43.1
decision equality*	…equal decision-making power between women and men in the household.*	35.2	39.6	34.7	36.5

*Statements marked with an asterisk (*) carried a negative connotation in relation to women’s rights as presented in the survey. However, for the purposes of this table, the corresponding SRHR concepts are framed in a rights-promotive manner to enhance clarity. For analysis, the Likert scale for these items had the highest score at ‘totally disagree’, whereas the positively connotated items had the highest score at ‘totally agree’.

A broadly consistent pattern emerged across provinces regarding which rights were perceived as most and least supported. The right to education, along with rights to relational and bodily autonomy, were most frequently endorsed, with 60–70% of respondents indicating perceived normative support. Respondents in Luanda generally reported higher levels of support than those in Huambo and Lunda Sul.

Approximately half of respondents indicated perceived normative support for the right to a life free from violence and for the ability to speak about experiences of violence without stigma. This was relatively consistent across provinces.

The most pronounced regional differences were observed for reproductive health and rights. In Lunda Sul, only 23.8% of respondents reported perceived normative support for delaying childbearing until at least 18 years of age, compared with 46.8% in Huambo and 61% in Luanda. A similar gradient was observed for recognition of female identity beyond motherhood: 31.6% of respondents in Lunda Sul reported that their communities valued women beyond maternal roles, compared with 47.4% in Huambo and 61.9% in Luanda. Support for limiting childbearing to protect women’s health was moderate, with just over half of respondents in Huambo and Luanda, and fewer than 40% in Lunda Sul, reporting normative support.

Intersecting topics relating to gender equality within intimate relationships—such as freedom of expression, consent within marriage, and shared decision-making—were consistently the least endorsed. Across all provinces, fewer than half of respondents reported that these principles were widely accepted within their communities.

### Psychometric assessment of the questionnaire

Most participants completed the gender norms questionnaire in full. Only a small proportion had one or two missing responses: 22 out of 910 in Luanda, 38 out of 684 in Huambo, and 7 out of 487 in Lunda Sul.

Application of the polytomous Rasch model to the original 5-point Likert scale revealed no evidence of strong structure in the residuals or local dependence (An additional file shows this in more detail [see Additional file 4]). Some item misfit was observed for bodily autonomy, freedom from violence, identity diversity, marital consent, and decision equality, with marital consent showing underfit: respondents with higher total scores tended to endorse this item less than expected [see Additional file 4 ]).

**Fig. 1:**
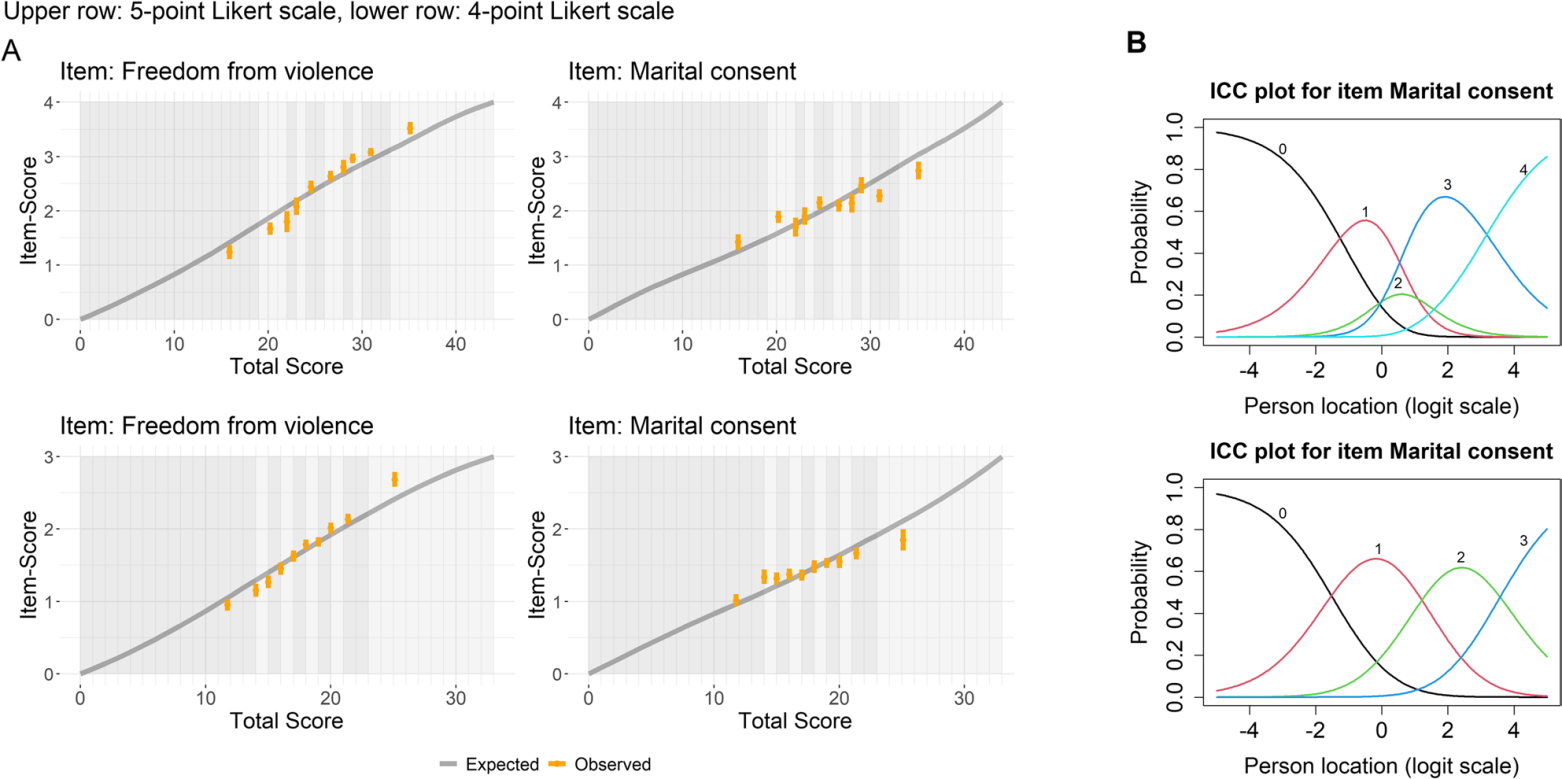
Results from the Rasch analysis illustrating the effect of changing the gender norms questionnaire’s original 5-point Likert scale (upper row) to a 4-point scale (lower row). **A:** Conditional item characteristic curve for an overfitting item (freedom from violence) and an underfitting item (marital consent). **B:** Item characteristic curves for marital consent as a representative example of all items (the disordering and consequent rectification appeared the same for all items). Analysis is based on data from the cross-sectional study with young women in Angola in 2022

A primary concern was the functioning of the middle response category, “it depends (neither agree nor disagree)”. This option was never the most probable response at any point along the latent continuum for any item, indicating disordered category thresholds (Fig. 1, and [Additional file 4]). Although ordered thresholds are not a strict requirement of the polytomous Rasch model [93], they are generally preferable from a measurement perspective, as they enhance confidence in the appropriateness and interpretability of the response categories. To address this, and given the limited conceptual alignment of the middle option with endorsement of fundamental human rights, the category was merged with the adjacent lower option, reducing the original five-point scale to four points. Empirically, the middle option was used sparingly: 77% of participants in Luanda, 78% in Huambo, and 80% in Lunda Sul selected it for no more than two items. The highest proportions occurred in relation to maternal health in Huambo (25%), marital consent in Luanda (21%), and bodily autonomy in Lunda Sul (20%).

**Fig. 2:**
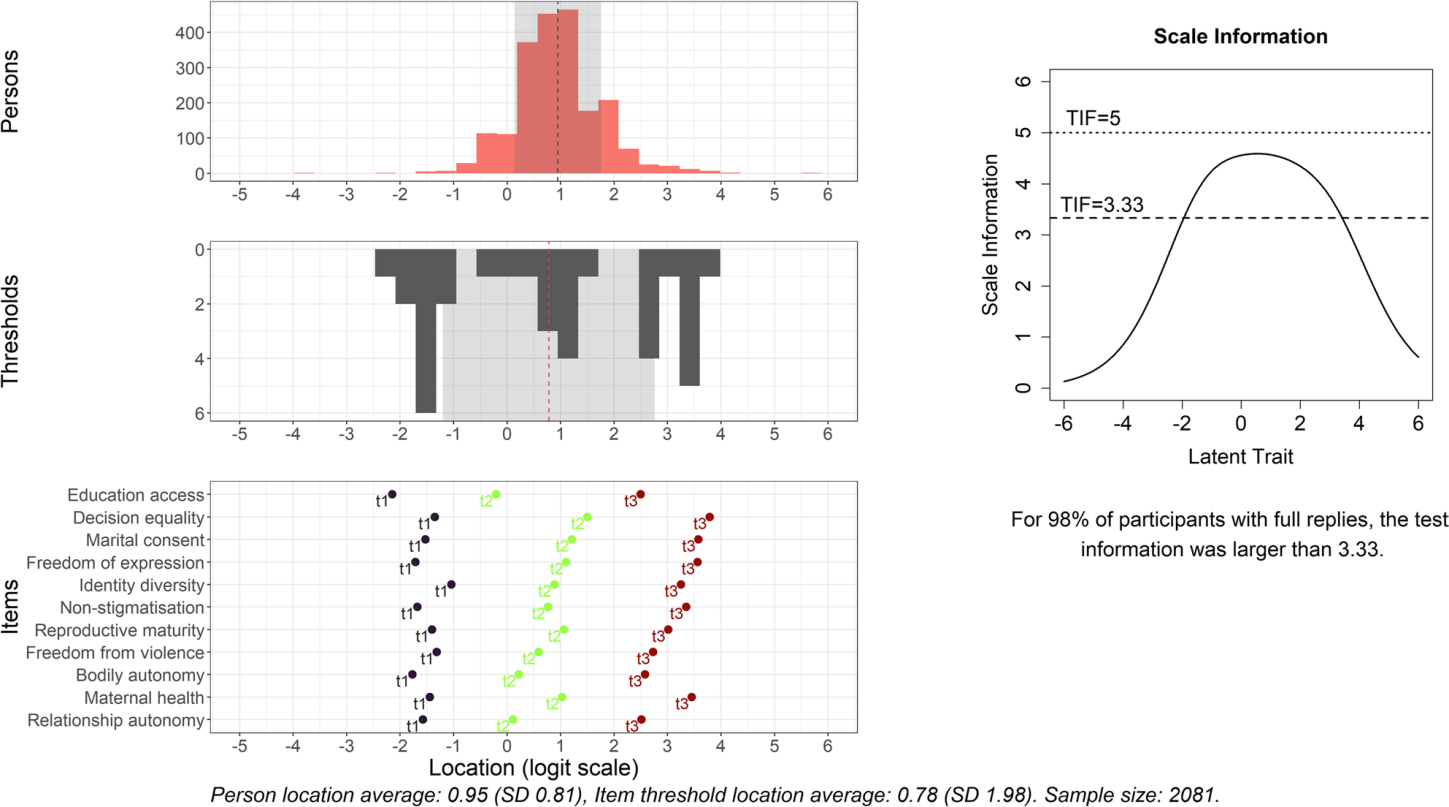
**Left:** Histograms of estimated person locations and item threshold locations for the gender norms questionnaire. A similar range and spread of these values are desirable for a good targeting. The positioning of item thresholds for each item is also shown (bottom plot). **Right:** Test information function for assessing reliability. Results are from the Rasch analysis based on data from the cross-sectional study with young women in Angola in 2022

Reanalysis using the modified 4-point scale improved category ordering and overall item fit [see Additional file 4]. Marital consent, however, continued to discriminate slightly less effectively than predicted (Fig. 1). Reducing the number of response categories decreased thresholds per item and introduced two broader gaps along the latent trait continuum (Fig. 2). For over 98% of participants with complete data, the test information function remained above 3.33 (Fig. 2), indicating fair reliability. The maximum test information did not exceed 5 (corresponding to a person separation index of 0.8), reflecting the relatively large uncertainties in person location estimates. The middle 50% of respondents were estimated between 0.43 and 1.46 on the latent scale, with an uncertainty of approximately 0.5 for each estimate.

**Fig. 3:**
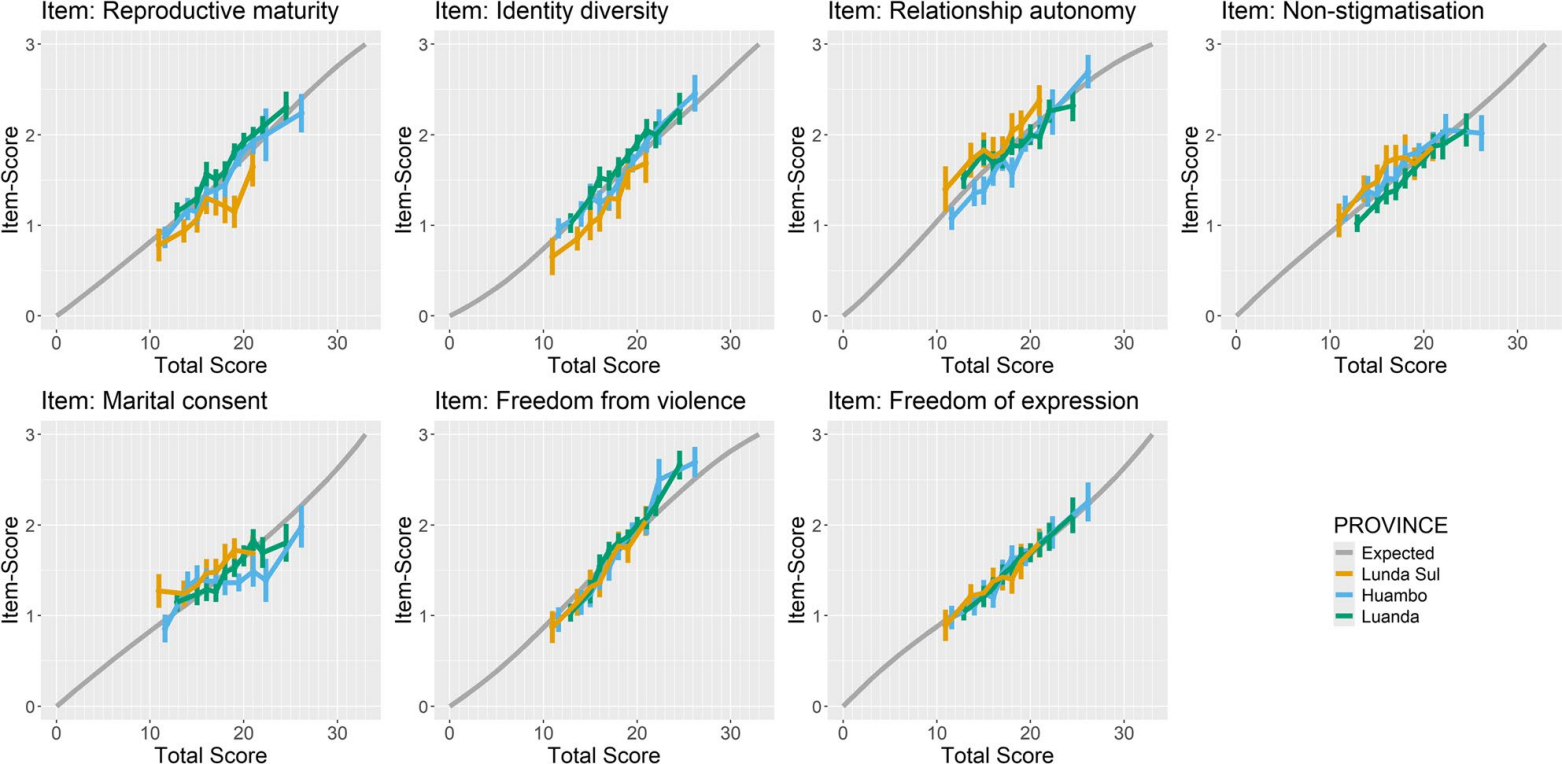
Selected differential item functioning plots for the gender norms questionnaire, contrasting the conditional expected scores from the common model (conditional on total scores) with the observed mean scores and their 95% confidence intervals in the provinces of Lunda Sul, Huambo, and Luanda. Differences in item functioning appear largest for reproductive maturity and identity diversity. The item marital consent shows a general misfit to the model, especially in Huambo. Freedom from violence and freedom of expression appear to function similarly across all provinces. Results are from the Rasch analysis based on data from the cross-sectional study with young women in Angola in 2022.

DIF largely corresponded with provincial divisions (see Additional file 4). Examination of DIF plots (Fig. 3) indicated that identity diversity and reproductive maturity were endorsed less frequently in Lunda Sul than expected based on total scores, whereas relationship autonomy and non-stigmatisation showed a slight opposite trend. Marital consent also displayed misfit in Huambo: respondents appeared reluctant to endorse the item despite higher total scores, i.e. scores indicating perceptions of relatively supportive community gender norms.

The impact of DIF on person location estimates was minimal, with differences remaining within the standard deviation of estimates [see Additional file 4]. Given the evidence of DIF across provinces, model fit was also examined separately stratified by province using the partial credit model. Results were satisfactory and broadly consistent with the overall analysis. Some variation in item hierarchy between provinces was observed [see Additional file 4]; however, the three lowest-positioned (“easiest”) items were consistent across provinces and aligned with the first three items listed in Table 3.

## Discussion

### Key findings

This study makes three principal contributions. First, it confirms the centrality of gender norms as an organising framework for SRHR in the Angolan context, while demonstrating the feasibility of developing questionnaire measures that both align with global policy priorities and resonate with local realities. Second, it shows that fewer than half perceived normative support for most of the examined SRHR, with endorsement for education and relationship autonomy appearing more stronger than that of reproductive rights or relational equality. Thirdly, the study highlights marked provincial differences in normative support for SRHR, reflecting wider socio-economic disparities. These findings indicate that young women’s capacity to assert and exercise SRHR is shaped not only by prevailing gender norms but also by the geographical and socio-economic contexts in which they are situated. Taken together, the results underscore the importance of combining validated measurement tools, which allow for cross-context comparability, with context-sensitive mapping methods—including participatory and qualitative approaches—and of tailoring interventions to local normative and structural realities.

### Capturing both visible and hidden norms

Several SRHR topics emphasised in international frameworks were also perceived as normatively salient in the Angolan context [1–8, 29, 30]. During questionnaire development, participants consistently prioritised bodily and relational autonomy, freedom from violence, equality in decision-making, and reproductive maturity, which were therefore incorporated. This alignment with theoretical expectations supports the measure’s construct validity [23, 24]. Rasch analysis further indicated that item difficulty broadly matched with participants’ perceptions of normative support. The absence of floor or ceiling effects suggests that the questionnaire was well calibrated to the study population, while the lack of item clustering supports the attempted coherence around a single latent construct [89]. Collectively, these results demonstrate the value of combining theoretical grounding with contextual adaptation to produce measures that are both locally meaningful and globally comparable.

Not all topics, however, could be operationalised. Some internationally recognised topics did not translate into items that resonated locally. Key informants and workshop participants often framed barriers to SRHR education and services as matters of resource scarcity rather than explicitly gendered issues. Yet the persistent underinvestment in maternal healthcare may itself reflect societal valuations of women’s roles [8, 40, 52], illustrating the difficulty of disentangling proximal from distal determinants of norms and entitlements [2, 11, 14, 18]. Highlighting women’s needs in contexts of widespread deprivation can also be sensitive, as claims may be construed as competing with those of other groups [23, 55]. These dynamics underline the policy relevance of multisectoral approaches to gender equity, which address the material as well as social conditions through which norms are sustained and contested [1–9, 23, 55, 59–62].

Further complexity emerged around gender identity and sexual orientation. Gender identity was most often articulated through widespread expectations surrounding motherhood, while other topics were described as socially invisible or rarely voiced. Such silences highlight how gendered inequities are reproduced differently across groups, with minority genders and sexualities facing risks of compounded marginalisation [1, 23]. At the same time, the narrow association between femininity and motherhood constrains all women’s capacity to inhabit alternative roles and identities [23, 25]. The extent to which women’s value is recognised beyond motherhood may therefore serve as a partial, albeit incomplete, indicator of openness to diversity in gender identities and expressions.

These challenges highlight the tension between the universality sought in international frameworks, the contextual specificity required for local relevance, and the imperative to attend to marginalised aspects of SRHR [5]. They demonstrate the importance of examining not only what is captured in survey data but also to what remains absent. Topics that prove difficult to operationalise should not be dismissed as technical omissions but recognised as possible reflections of stigma, silence, or contextual particularity [1, 23]. Limitations in representing the multiple facets of gender equality and equity are, however, not confined to local contexts; international frameworks themselves may embody conceptual blind spots, particularly in under-researched settings such as Angola [8]. Combining standardised instruments with qualitative approaches that build trust and enable deeper reflection is therefore not merely supplementary but, in some cases, a necessary precursor to quantitative measurement [1, 24].

### Navigating normative tensions in support for SRHR

The study identified both convergences and divergences in normative support. Descriptive analyses and Rasch modelling revealed consistent patterns. When grouped thematically [1, 5], sexual rights appeared more strongly supported than reproductive rights. Endorsement of relationship and bodily autonomy approached 70% across the study population, compared with around 50% for reproductive rights such as reproductive maturity and maternal health.

Weak support for reproductive maturity may reflect limited recognition of girls’ entitlement to an autonomous and protected childhood, coupled with enduring expectations that young women prioritise reproductive roles [76]. Normative assumptions that position women’s primary value in terms of reproduction may also contribute to the marginalisation of men’s participation in family planning and maternal health [13, 14]. These findings indicate that many young women are expected to assume caregiving responsibilities early in life, often without corresponding decision-making power or adequate structural support. While these roles may provide meaning and purpose, they also risk placing disproportionate emotional and practical burdens on young women, constraining the capacity to exercise SRHR and to pursue long-term educational or socio-economic aspirations [1, 23]. These normative expectations were compounded by structural deficits, including insufficient maternal health resources, uneven access to pregnancy-related information (reported by nearly 70% of women in Luanda and Huambo, compared with only 30% in Lunda Sul), and material deprivation (with more than 40% reporting severe food shortages within their household). Taken together, these dynamics point to a disconnect between prevailing normative frameworks and the structural conditions required to safeguard women’s health and family well-being [18, 31, 32, 50–54].

By contrast, stronger support for certain sexual rights suggests that young women may experience a degree of agency in choosing whether—and with whom—to enter relationships. This offers potential entry points for rights-based interventions [3, 5, 25, 46–51]. Yet such opportunities are complicated by ambivalence around IPV. In Lunda Sul, for example, nearly 70% affirmed community support for women’s rights to choose a partner, but fewer than half perceived support for women leaving violent relationships, and more than two-thirds reported experiencing IPV themselves. This paradox reveals a crucial policy challenge: young women may be recognised as entitled to choose a partner, but not fully supported in leaving harmful relationships. Addressing this gap requires not only reinforcing sexual rights but also integrating IPV prevention into such strategies—an engagement with the contradictions embedded in prevailing gender norms [8, 30].

Despite formal commitments to gender equality, the findings indicate that many young women still inhabit contexts where key rights lack normative legitimacy. Items on intimate partnerships showed only modest support (35–50%) for rights such as freedom of expression, refusal of sex within marriage, decision-making equality, and the ability to limit pregnancies for health reasons. These findings provide another illustration of enduring expectations that women should prioritise the needs of others [29, 55]. Psychometric results reinforced this: in Huambo and Luanda, marital sexual consent and equality in decision-making emerged as the most difficult items for respondents to endorse as socially legitimate, signalling hesitation or resistance. These findings highlight the underlying social tensions and contestations, revealed through Rasch analyses.

Taken together, this illuminates the interwoven symbolic and structural dimensions of motherhood and partnership, and the ongoing negotiation between long-standing gender roles and aspirations for equality [8–10, 28]. Addressing these tensions requires cultural sensitivity and recognition that established norms provide meaning and social cohesion while also restricting rights and opportunities. For policy, this means striking a balance: reducing inequalities and challenge discriminatory practices, while acknowledging the social fabrics through which communities sustain identity, well-being and resilience [8, 10, 59, 77–84].

### Contextual variation in normative SRHR support

The observed variations in normative support for SRHR extended beyond thematic distinctions, revealing pronounced subnational disparities. While some aspects—such as access to education, non-stigmatisation, equality in decision—elicited relatively consistent support across provinces, other topics diverged sharply. For instance, around 60% of respondents in Luanda stated normative support for reproductive maturity and identity diversity, compared with only 20–30% in Lunda Sul, with Huambo occupying an intermediate position.

Although it was beyond the scope of this study to examine associations between broader SRHR characteristics and the prevalence of normative support, prior research underscores the influence of social norms on behaviour, decision-making, and self-perception, often operating through mechanisms of collective approval and sanction [9, 22, 24, 35, 57]. It is therefore unsurprising that early pregnancies were more frequently reported in provinces with lower support for reproductive maturity—around 50% of respondents in Lunda Sul and Huambo, compared with about one-fifth in Luanda, had experienced pregnancy before the age of 18.

The Rasch model further illuminated provincial variation in item performance. Most items fitted well, but some proved difficult to endorse. In Lunda Sul, reproductive maturity and identity diversity were particularly difficult to affirm as socially legitimate, and DIF analyses confirmed systematically lower endorsement compared with the other provinces, even among respondents with similar overall scores. In Huambo, items on marital consent underperformed: even those reporting overall strong community support for SRHR expressed hesitancy here. Such patterns point to context-specific conceptual ambiguities or deeply embedded social norms, highlighting the importance of further inquiry into historical, legal and cultural narratives shaping these constructs [8–12, 30].

In a similar vein, although DIF did not substantially distort total scores, its presence indicates that aggregate results may conceal meaningful differences in how normative beliefs are configured across settings. This has direct policy implications: one high overall score may reflect strong support for particular SRHR topics, while another may indicate endorsement of quite different topics—two similar totals that nonetheless signal distinct emphases within the SRHR field and may carry different health implications [9, 22].

These findings underscore the complementary value of both summary indicators and disaggregated analyses. Composite scores offer a broad overview of the normative climate, but disaggregated analyses are essential for identifying which rights are more, or less, socially legitimised in a given context. Rasch modelling was particularly valuable in this regard, revealing items that were locally difficult to endorse (e.g., reproductive maturity and identity diversity in Lunda Sul) as well as those with unexpected response patterns (e.g., marital consent in Huambo). Such analyses illustrate the utility of modern psychometric tools in uncovering not only the strength but also the texture of normative support, including intra-country variation.

The policy significance of this approach lies in its ability to inform interventions responsive to specific local conditions. Regional differences in SRHR support largely reflected disparities in socio-economic development and access to services, as seen in national statistics [32, 40, 42] and corroborated by this study. In Lunda Sul and Huambo, where normative SRHR support was weakest, respondents also reported high levels of economic insecurity, with nearly all situated in the two lowest wealth tertiles. In Lunda Sul, structural constraints were acute: while nearly 70% of participants in Luanda and Huambo reported menstrual autonomy and correctly identified the fertile period, this applied to only about 30% in Lunda Sul. These disadvantages coincided with high rates of under-age pregnancy and IPV—affecting more than half of respondents in Lunda Sul and nearly half in Huambo, compared to about one-fifth and two-fifths, respectively, in Luanda. Considered alongside documented gaps in family planning and maternal health services [6, 7, 30, 32, 40], these findings highlight how normative and structural barriers may intersect and mutually reinforce one another to constrain SRHR [2, 5, 81].

The convergence of restrictive norms with structural disadvantage aligns with global evidence that gender norms often intersect with socio-economic marginalisation, compounding risks and vulnerabilities [1–3, 23, 25]. It also resonates with intersectional frameworks long advanced by the Angolan women’s movement, which have situated gender inequality within broader struggles against economic injustice, ethnic discrimination, and colonial legacies [8, 27–30, 77]. From this perspective, gender norms cannot be meaningfully understood in isolation from the wider systems of power and exclusion shaping everyday life. While no single measurement tools can capture these intersections in full, acknowledging them strengthens the rationale for contextually grounded and socially responsive SRHR strategies [2, 10, 20, 25, 79, 83].

### Limitations and generalisability

This study benefited from a high response rate, the inclusion of participants from diverse backgrounds, and a study design carefully adapted to the local context and rigorously pre-tested. Nevertheless, several limitations must be acknowledged to ensure a balanced interpretation of the findings.

The use of self-reported data collected through interviews enabled exploration of young women’s perspectives on normative beliefs within a safe setting, while nonetheless carrying an inherent risk of social desirability bias. The focus on a specific age cohort and a non-random sampling strategy also constrains generalisability. That said, the demographic profile of the sample broadly aligns with comparable studies and reflects known national and provincial socio-economic patterns [32, 40–43, 73], suggesting transferability to similar settings.

The tailored questionnaire represents an important step in measuring gender norms related to SRHR in Angola, but also carries limitations. While it accounted for socio-economic and regional variation, it did not fully engage with the intersections of systemic inequality, risking an analytical stance that treats gender as an isolated factor. Scholars have emphasised the importance of situating gender within wider socio-economic and political structures [8, 78–84]. Critical and postcolonial perspectives further caution against universalist assumptions and Eurocentric epistemologies that risk obscuring the complexity of lived experiences [14, 27, 78–82]. This study seeks to contribute to a more multifaceted understanding of gender norms, though ultimately it is for the reader to judge its success.

The reliance on binary gender categories presents a limitation. By classifying participants as ‘women’ and ‘men’, the study risks reproducing reductive assumptions [23]. Neither the sampling strategy nor the questionnaire explicitly accounted for the diversity of gender identities. Items fully addressing gender fluidity were not successfully developed, and reliance on a single proxy question constrains insights. A fuller understanding requires in-depth research into diverse gender expressions, including identities beyond those linked to parenthood. Nevertheless, in contexts where expectations of early pregnancy and high fertility intersect with maternal mortality and limited maternal care [5, 8, 33, 34, 40], research focusing on individuals assigned female at birth remains essential, while recognising that gendered experiences are never monolithic.

Methodologically, some measurement challenges also warrant consideration. Although the questionnaire showed acceptable psychometric properties after response-scale adjustments, DIF across provinces and only moderate test information values raise questions about precision. Future work could explore more nuanced tools, potentially organised into subscales, to enhance analytical specificity and support monitoring, policy, and intervention design.

The middle response category (‘it depends’) posed difficulties: it was too rarely selected to stand apart and was therefore merged with an adjacent category. This decision was justified on the grounds that hesitation regarding core SRHR topics—such as maternal health, marital sexual consent, and bodily autonomy—likely reflect rights-negative orientations. Yet such responses may also convey meaningful ambivalence, which are often central to how norms are negotiated. As the same pattern may not recur in other samples [64], caution is warranted: retaining the category could provide insights into contentious or ambiguous items. Further exploration of how respondents interpret this option would be valuable. Psychometric alternatives, such as alternative IRT models [102], might address threshold issues but could reduce compatibility with established measurement principles [103].

Together, these limitations highlight the need for continued refinement of measurement tools, and for participatory, intersectional and context-sensitive approaches in SRHR research. They also point to critical avenues for future investigation. Readers are encouraged to interpret the findings with these considerations in mind.

## Conclusions

The study demonstrates that participatory mixed-methods approaches can facilitate the identification of SRHR topics that are at once aligned with global normative debates and perceived as meaningful within local contexts. The participatory design also supported the translation of these topics into a questionnaire accessible to a population characterised by diverse educational backgrounds and living conditions.

A central contribution lies in the finding that the identified topics together can be viewed as reflecting a single latent construct, enabling future applications to capture overall patterns of support or constraint regarding SRHR within the study population. At the same time, the analysis highlighted minor tensions around specific items, underscoring the importance of retaining item-level analyses and remaining attentive to locally embedded dynamics. These findings point to the need for further inquiry across diverse contexts and population groups before consensus can be reached on how SRHR-related gender norms might most appropriately be measured. Continued investigation accords with the understanding that sustainable and transformative development requires approaches that are contextually grounded, locally resonant, and attuned to structural dimensions of inequality and justice.

Future research and practice may benefit from a two-step strategy. When applying the questionnaire beyond the present study population, the initially identified SRHR topics (Table 1) could serve as a point of departure for focus group discussions to adapt content to new contexts. This exploratory phase may also generate additional questions that capture context-specific nuances not fully represented in the current framework. In a subsequent phase, psychometric analyses can be conducted at the item level—both for the items tested in this study and for newly developed ones—allowing comparability across contexts while maintaining sensitivity to local particularities. Where resources do not permit Rasch analyses, item-level data may nevertheless provide valuable descriptive insights into patterns of normative support and inform the design of SRHR-promoting action.

In summary, the study suggests that while certain dimensions of gender norms resonate across contexts, they are also embedded in historical, social, and material conditions. This underlines the value of participatory and reflexive approaches that engage with specific social and institutional structures, while simultaneously fostering individual agency and strengthening rights-based systems. Such strategies hold promise not only for advancing SRHR but also for promoting dignity and equity more broadly.

It is concerning that many of the participating young Angolan women encounter substantial challenges in their everyday lives, and that only a limited range of SRHR topics were perceived by a majority as enjoying broad societal acceptance. Even so, the relative convergence between international and national actors’ perspectives on young women’s SRHR as a priority offers cautious grounds for optimism that sustained collaborative efforts may contribute to conditions in which SRHR are not only formally inscribed in policy but also meaningfully realised in young women’s lived experiences.

## Supplementary Information

Supplementary Material 1.

Supplementary Material 2.

Supplementary Material 3.

Supplementary Material 4.

Supplementary Material 5.

## Data Availability

The qualitative interview data generated during the current study are not publicly available in order to protect participant confidentiality. All quantitative data generated or analysed during this study are included in this published article and its supplementary information files.

## Abbreviations

CICC: Conditional Item Characteristic Curve
CSO: Civil Society Organisation
DHS: Demographic Health Survey
DIF: Differential Item Functioning
IPV: Intimate Partner Violence
SADIMA: SAúde e SIreitos das Mulheres em Angola (Angolan women’s health and rights)
SRHR: Sexual and Reproductive Health and Rights

## References

[1] Starrs AM, Ezeh AC, Barker G, Basu A, Bertrand JT, Blum R, et al. Accelerate progress-sexual and reproductive health and rights for all: report of the Guttmacher-Lancet commission. Lancet. 2018;391(10140):2642–92. 29753597 10.1016/S0140-6736(18)30293-9

[2] World Health Organization (WHO). World report on social determinants of health equity. Geneva: World Health Organization. 2025. Licence: CC BY-NC-SA 3.0 IGO. https://www.who.int/publications/i/item/9789240107588. Accessed 31 May 2025.

[3] United Nations (UN). The sustainable development goal report 2023: Special edition. Towards a rescue plan for people and planet. https://sdgs.un.org/sites/default/files/2023-07/The-Sustainable-Development-Goals-Report-2023_0.pdf. Accessed 17 June 2025.

[4] African Union (AU). Protocol to the African charter on human and peoples’ rights on the rights of women in Africa. African Union. 2003. https://au.int/sites/default/files/treaties/37077-treaty-charter_on_rights_of_women_in_africa.pdf. Accessed 17 June 2025.

[5] Kågesten A, Båge K, Sundewall J, Litorp H, Puranen B, Uthman O et al. Sexual and reproductive health and rights: Measuring values and norms to guide Swedish development cooperation, EBA report 2021:04, The expert group for aid studies (EBA), Sweden. https://www.researchgate.net/publication/356754461_Sexual_and_Reproductive_Health_and_Rights_Measuring_Values_and_Norms_to_Guide_Swedish_Development_Cooperation. Accessed 22 June 2025.

[6] Governo de Angola. Plano de desenvolvimento nacional, 2023-27 (PDN 2023–2027). Impacto sócioeconómico sustentável. Accessed 17 June 2025.

[7] União Europeia (EU) Diagnóstico igualidade de género Angola. 2022. Facilidade de diálogo UE-Angola. https://www.eeas.europa.eu/sites/default/files/documents/Diagn%C3%B3stico%20da%20Igualdade%20de%20G%C3%A9nero%20em%20Angola%202022.pdf. Accessed 17 June 2025.

[8] Mouzinho Â, Cutaia S. Reflections on feminist organising in Angola. Feminist Afr 2017 Dec 1(22):33–51.

[9] United Nations Development Programme (UNDP). Breaking down gender biases: shifting social norms towards gender equality. 2023 gender social norms index. New York: UNDP; 2023. https://www.undp.org/sv/sweden/publications/2023-gender-social-norms-index-gsni. Accessed 17 20025.

[10] Cislaghi B, Heise L. Gender norms and social norms: differences, similarities and why they matter in prevention science. Sociol Health Illn. 2020;42(2):407–22. 10.1111/1467-9566.13008. 31833073 10.1111/1467-9566.13008 PMC7028109

[11] United Nations Development Programme (UNDP). Tackling social norms: A game changer for gender inequalities. 2020 Human Development Perspectives. New York: UNDP. 2020. https://www.undp.org/arab-states/publications/tackling-social-norms-game-changer-gender-inequalities. Accessed 17 June 2025.

[12] Inglehart R, Norris P. Rising tide: gender equality and cultural change around the world. Cambridge: Cambridge University Press; 2003.

[13] Davis SN, Greenstein TN. Gender ideology: Components, predictors, and consequences. Annu Rev Sociol. 2009;35:87–105.

[14] Connell R. Rethinking gender from the South. Feminist Stud. 2014;40(3):518.

[15] Horne C, Mollborn S. Norms: an integrated framework. Annu Rev Sociol. 2020;46:467–87. 10.1146/annurev-soc-121919-054658.

[16] Gelfand MJ, Gavrilets S, Nunn N. Norm dynamics: interdisciplinary perspectives on social norm emergence, persistence, and change. Annu Rev Psychol. 2024;75:341–78. 10.1146/annurev-psych-033020-013319. 37906949 10.1146/annurev-psych-033020-013319

[17] Chirowa F, Atwood S, van der Putten M. Gender inequality, health expenditure and maternal mortality in sub-Saharan africa: A secondary data analysis. Afr J Prim Healthc Fam Med. 2013;5(1):1.

[18] Clark J, Horton R. A coming of age for gender in global health. Lancet. 2019;393(10189):2367–9. 10.1016/S0140-6736(19)30986-9. 31155274 10.1016/S0140-6736(19)30986-9

[19] Loll D, Fleming PJ, Manu A, Morhe E, Stephenson R, King EJ, et al. Reproductive autonomy and pregnancy decision-making among young Ghanaian women. Glob Public Health. 2020;15(4):571–86. 10.1080/17441692.2019.1695871. 31766950 10.1080/17441692.2019.1695871 PMC7093254

[20] Sedlander E, Bingenheimer JB, Long MW, et al. The G-NORM scale: development and validation of a theory-based gender norms scale. Sex Roles. 2022;87:350–63. 10.1007/s11199-022-01281-8. 36168556 10.1007/s11199-022-01319-9 PMC9508194

[21] Svallfors S, Båge K, Ekström AM, Dessie Y, Wado YD, Fagbemi M, et al. Support for sexual and reproductive health and rights in Sub-Saharan africa: a new index based on world values survey data. Reproductive Health. 2024;21(1):90–12. 38918832 10.1186/s12978-024-01820-2 PMC11197335

[22] McCook S, Powell A. (2020). Feasibility study into the possible inclusion of social norms measures within the 2021 National Community Attitudes towards violence against women Survey (NCAS) (Research report, 02/2020). Sydney, NSW: ANROWS. https://www.anrows.org.au/publication/feasibility-study-into-the-possible-inclusion-of-social-norms-measures-within-the-2021-ncas-research-report. Accessed 17 June 2025.

[23] Sundewall J, Båge K, Ekström AM, Puranen B, Litorp H, Uthman OA, et al. Addressing the second ‘R’ in sexual and reproductive health and rights: why norms and values matter for development Cooperation. BMJ Global Health. 2022;7:e008520. 10.1136/bmjgh-2022-00852. 35654447 10.1136/bmjgh-2022-008520 PMC9163001

[24] Gupta GR, Oomman N, Grown C, Conn K, Hawkes S, Shawar YR, et al. Gender equality and gender norms: framing the opportunities for health. Lancet. 2019;393(10190):2550–62. 10.1016/S0140-6736(19)30651-8. 31155276 10.1016/S0140-6736(19)30651-8

[25] Weber AM, Cislaghi B, Meausoone V, Abdalla S, Mejía-Guevara I, Loftus P, et al. Gender norms and health: insights from global survey data. Lancet. 2019;393(10189):2455–68. 10.1016/S0140-6736(19)30765-2. 31155273 10.1016/S0140-6736(19)30765-2

[26] Candido MP. Women in Angola. In Oxford research encyclopedia of African history. 2018. 10.1093/acrefore/9780190277734.013.569

[27] Telo FCA. A pós/decolonialidade e os movimentos de mulheres e feministas na África, in: Garcia MdF and da Silva JAN. Africanidades, afrobrasilidade e process (des)colonizador: contribuicoes à implementacao da Lei 10.639/03. Universidade federal da Paraíba. 2018. https://www.editora.ufpb.br/sistema/press5/index.php/UFPB/catalog/book/69. Accessed 17 June 2025.

[28] Strippoli G. Women’s transnational activism against portugal’s colonial wars. Int Rev Soc Hist. 2022;67(S30):209–36. 10.1017/S0020859022000037.

[29] Telo FCA. Direitos reprodutivos em Angola: A utopia does direitos ou o direito à utopia? in: Ferreira LFG, de Moura LLD, Franca MHO, Araújo MMB. ANAIS: IX Seminário internacional de direitos humanos da UFPB. Desafios e perspectivas da democracia na América Latina. 2017. http://www.cchla.ufpb.br/ncdh/wp-content/uploads/2017/09/IX-SIDH_Anais-Eletr%C3%B4nicos-2.pdf. Accessed 17 June 2025.

[30] Mosaiko. Relatório da pesquisa sobre políticas públicas inclusivas numa perspectiva de género, 2019–2021, Luanda: Tipografia Coimbra. 2021. https://mosaiko.op.org/wp-content/uploads/2019/05/PAPPIA-Relatorio-de-Pesquisa-Web.pdf. Accessed 17 June 2025.

[31] World Health Organization (WHO). Trends in maternal mortality 2000 to 2020: estimates by WHO, UNICEF, UNFPA, World Bank Group and UNDESA/Population Division., Geneva: World Health Organization, Licence: CC BY-NC-SA 3.0 IGO; 2023. https://www.who.int/publications/i/item/9789240068759. Accessed 17 June 2025.

[32] Instituto Nacional de Estatística (INE), Minstério da Saúde (MINSA), Ministério do Planeamento e do Desenvolvimento Territorial (MINPLAN), ICF. Angola inquérito de indicadores múltiplos e de saúde (IIMS) 2015–2016. 2017. https://www.dhsprogram.com/pubs/pdf/SR238/SR238.P.pdf. Accessed 17 May 2025.

[33] Umar AS, Kabamba L. Maternal mortality in the main referral hospital in Angola, 2010–2014: Understanding the context for maternal deaths amidst poor Documentation. Int J MCH AIDS. 2016;5(1):61–71. 28058194 10.21106/ijma.111 PMC5187641

[34] Rosário EVN, Gomes MC, Brito M, Costa D. Determinants of maternal healthcare and birth outcome in the dande health and demographic surveillance system area, Angola. PLoS ONE. 2019;14(8):e0221280. 31437180 10.1371/journal.pone.0221280 PMC6706050

[35] de Almeida N, Teixeira A, Capoco Sachiteque A, Molina JR, dos Prazeres Tavares H, Ramalho C. Characterisation of induced abortion and consequences to women’s health at hospital central do Huambo-Angola. J Obstet Gynaecol. 2020;40(4):558–63. 31475598 10.1080/01443615.2019.1635096

[36] Aoki A, Mochida K, Kuramata M, Sadamori T, Sapalalo P, Tchicondingosse L, et al. Association between the quality of care and continuous maternal and child health service utilisation in angola: longitudinal data analysis. J Glob Health. 2023;13:04073. 37565413 10.7189/jogh.13.04073 PMC10416139

[37] UN Women. The Women Count Data Hub, Angola. https://data.unwomen.org/country/angola. Accessed 17 May 2025.

[38] Prata N, Bell S, Fraser A, Carvalho A, Neves I, Nieto-Andrade B. Partner support for family planning and modern contraceptive use in Luanda, Angola. Afr J Reprod Health. 2017;21(2):35–48. 29624938 10.29063/ajrh2017/v21i2.5

[39] Yaya S, Ghose B. Prevalence of unmet need for contraception and its association with unwanted pregnancy among married women in Angola. PLoS ONE. 2018;13(12):e0209801. 30596733 10.1371/journal.pone.0209801 PMC6312300

[40] Instituto Nacional de Estatística (INE), Minstério da Saúde (MINSA), Inner City Fund (ICF). 2024. Inquérito de indicadores múltiplos e de saúde de Angola, 2023–2024: relatório de indicadores básicos. https://www.ine.gov.ao/Arquivos/arquivosCarregados/Carregados/Publicacao_638487571879652908.pdf. Accessed 17 May 2025.

[41] dos Reis FVD, Macama A, Schjøtt S, Laflamme L, Kessel B, Priebe GE. Health literacy and institutional delivery among young Angolan women: a cross-sectional study in three provinces, medrxiv: 25323791 [Preprint]. 2025 [cited 2025-03-11]. 10.1101/2025.03.11.25323791

[42] Pacatolo C, Boio D, Kitombe C. Angolans dissatisfied with government efforts to promote equal rights for women. Afrobarometer Dispatch No 622, 28 March 2023. https://www.afrobarometer.org/wp-content/uploads/2023/03/AD622-Angolans-dissatisfied-with-government-performance-on-gender-equality-Afrobarometer-28march2023.pdf. Last accessed 17 May 2025.

[43] Skandro S, Abio A, Baernighausen T, Lowery Wilson M. Socio-demographic determinants of intimate partner violence in angola: a cross-sectional study of nationally representative survey data. Arch Womens Ment Health. 2024;27(1):21–33. 10.1007/s00737-023-01376-3. 37816985 10.1007/s00737-023-01376-3 PMC10791757

[44] World Health Organization (WHO). Global health observatory data repository. Maternal and reproductive health. https://data.who.int/indicators/i/BEDE3DB/E0D4E17. Accessed 17 May 2025.

[45] Sama CB, Ngasa SN, Dzekem BS, Choukem SP. Prevalence, predictors and adverse outcomes of adolescent pregnancy in sub-Saharan africa: a protocol of a systematic review. Syst Rev. 2017;6(1):247. 29208035 10.1186/s13643-017-0650-0 PMC5718137

[46] Olubodun T, Rahman SA, Odukoya OO, Okafor IP, Balogun MR. Determinants of health facility delivery among young mothers aged 15–24 years in nigeria: a multilevel analysis of the 2018 Nigeria demographic and health survey. BMC Pregnancy Childbirth. 2023;23(1):185. 36932391 10.1186/s12884-023-05492-x PMC10024451

[47] Asmamaw DB, Tafere TZ, Negash WD. Prevalence of teenage pregnancy and its associated factors in high fertility sub-Saharan Africa countries: a multilevel analysis. BMC Women’s Health. 2023;23(1):23. 36650514 10.1186/s12905-023-02169-7 PMC9843834

[48] Koerner E, Zandamela J, Duckett A. Menstrual management in Angola: effectiveness of providing quality menstrual products and educational workshops in Huambo, Huíla, Luanda and Lunda Sul. UNFPA Learning Study. 2021. https://angola.unfpa.org/sites/default/files/pub-pdf/ebe_girl_unfpa_angola_mh_study_phase_1_en_2021-05-24_final.pdf. Last accessed 17 May 2025.

[49] Oliveira D, de Oliveira JM, Martins MR, Barosso MR, Castro R, Cordeiro L, et al. Maternal profiles and pregnancy outcomes: a descriptive cross-sectional study from Angola. Matern Child Health J. 2023;27:2091–8. 37815656 10.1007/s10995-023-03782-6

[50] Silva MR, Roque HC, Caetano A. Culture in angola: insights for human resources management. Cross Cult Manag. 2015;22(2):166–86.

[51] Global Change Data Lab. Our world in data, Angola. 2022. https://ourworldindata.org/country/angola. Accessed 17 June 2025.

[52] World Bank Group. Gender Data Portal: Angola. https://genderdata.worldbank.org/en/economies/angola. Accessed 17 June 2025.

[53] Inter-Parliamentary Union (IPU). Angola: Data on women in parliament. IPU Parline-Global data on national parliaments. https://data.ipu.org/parliament/AO/AO-LC01/data-on-women/. Accessed 17 June 2025.

[54] World Economic Forum. (July 13, 2022). Gender gap index in Angola from 2016 to 2022 [Graph]. In *Statista*. https://www.statista.com/statistics/1220535/gender-gap-index-in-angola/. Accessed 17 June 2025.

[55] World Health Organization (WHO), United Nations Population Fund (UNFPA). Ending Preventable Maternal Mortality (EPMM). A renewed focus for improving maternal and newborn health and wellbeing. https://www.who.int/publications/i/item/9789240040519. Accessed 17 June 2025.

[56] Upadhyay UD, Karasek D. Women’s empowerment and ideal family size: an examination of DHS empowerment measures in Sub-Saharan Africa. Int Persp Sex Reprod Health. 2012;38(2):78–89. 10.1363/3807812 22832148

[57] Nartey P, Bahar OS, Nabunya P. A review of the cultural gender norms contributing to gender inequality in ghana: an ecological systems perspective. J Int Womens Stud. 2023;25(7):1–16. PMC11086636 38736590

[58] Hay K, McDougal L, Percival V, Henry S, Klugman J, Wurie H, et al. Gender equality, norms, and health steering committee. Disrupting gender norms in health systems: making the case for change. Lancet. 2019;393(10190):2535–49. 10.1016/S0140-6736(19)30648-8. 31155270 10.1016/S0140-6736(19)30648-8 PMC7233290

[59] Heise L, Greene ME, Opper N, Stavropoulou M, Harper C, Nascimento M, et al. Gender inequality and restrictive gender norms: framing the challenges to health. Lancet. 2019;393(10189):2440–54. 10.1016/S0140-6736(19)30652-X. 31155275 10.1016/S0140-6736(19)30652-X

[60] Heymann J, Levy JK, Bose B, Ríos-Salas V, Mekonen Y, Swaminathan H, et al. Improving health with programmatic, legal, and policy approaches to reduce gender inequality and change restrictive gender norms. Lancet. 2019;393(10190):2522–34. 10.1016/S0140-6736(19)30656-7. 31155271 10.1016/S0140-6736(19)30656-7

[61] Sen G, Ostlin P, George A. Unequal, unfair, ineffective and inefficient gender inequity in health: why it exists and how we can change it: final report to the WHO commission on social determinants of health. Arrows Change. 2007;13(1):9.

[62] Hammarström A, Hensing G. How gender theories are used in contemporary public health research. Int J Equity Health. 2018;17:34. 10.1186/s12939-017-0712-x. 29554916 10.1186/s12939-017-0712-x PMC5859645

[63] Miani C, Wandschneider L, Niemann J, Batram-Zantvoort S, Razum O. Measurement of gender as a social determinant of health in epidemiology-a scoping review. PLoS ONE. 2021;16(11):e0259223. 10.1371/journal.pone.0259223. 34731177 10.1371/journal.pone.0259223 PMC8565751

[64] Nanda G. Compendium of gender scales. Washington, DC: FHI 360/C-Change; 2011.

[65] Milner A, Kavanagh A, Scovelle AJ, O’Neil A, Kalb G, Hewitt B, King TL. Gender equality and health in high-income countries: a systematic review of within-country indicators of gender equality in relation to health outcomes. Womens Health Rep (New Rochelle). 2021;2(1):113–23. 10.1089/whr.2020.0114. 33937909 10.1089/whr.2020.0114 PMC8082013

[66] Knisely KA, Wind S. Gendered Language attitudes: exploring Language as a gendered construct using Rasch measurement theory. J Appl Meas. 2015;16(1):95–112. 25562339

[67] Andrich D, Marais I. A course in Rasch measurement theory. Measuring in the educational, social and health sciences. 1st ed. 2019. Singapore: Springer Nature; 2019.

[68] Stoebenau K, Kyegombe N, Bingenheimer JB, Ddumba-Nyanzi I, Mulindwa J. Developing experimental vignettes to identify gender norms associated with transactional sex for adolescent girls and young women in central Uganda. J Adol Health. 2019;64(4):S60–6. 10.1016/j.jadohealth.2018.11.009 PMC6426717 30914170

[69] Baidoo L. Bridging development interventions and women’s empowerment in ghana: reflections from radical feminist perspectives. Feminist Afr. 2022;3(2):79–97.

[70] Oliveira RS. Magnificent and beggar land: Angola since the civil war. Cary: Oxford University Press, Inc.; 2015.

[71] Global Change Data Lab. Our world in data, Angola. Data source: World Bank poverty and inequality platform 2024. https://ourworldindata.org/grapher/share-of-population-in-extreme-poverty-2011-ppp?tab=chart&country=AGO. Accessed 17 June 2025.

[72] World Bank. Angola poverty assessment report June 24, 2020, poverty and equity global practice, Africa region. https://documents1.worldbank.org/curated/en/328741593674436204/pdf/Angola-Poverty-Assessment.pdf. Accessed 17 June 2025.

[73] Catoto Capitango JA, Garat de Marin MS, Soriano Flores E, Rojo Gutiérrez MA, Gracia Villar M, Durántez Prados FÁ. Inequalities and asymmetries in the development of angola’s provinces: the impact of colonialism and civil war. Soc Sci. 2022;11(8):334. 10.3390/socsci11080334.

[74] Rodrigues CU, Bryceson DF. Precarity in Angolan diamond mining towns, 1920–2014: tracing agency of the state, mining companies and urban households. J Mod Afr Stud. 2018;56(1):113–41.

[75] Priebe GE, Macama A, Kessel B, dos Reis FVD, Van Dúnem JE, Katito J et al. Associations between social capital and other living conditions among young Angolan women: a cross-sectional study in three provinces. MedRxiv:25320530 [Preprint]. 2025 [cited 2025-01-20]. 10.1101/2025.01.14.25320530

[76] Associação Mwana Pwo. Apenas uma criança. Explorando casamento infantil em Angola. https://www.facebook.com/mwanapwo.org. Accessed 17 June 2025.

[77] Acção para o Desenvolvimento Rural e Ambiende (ADRA). A participação da mulher na produção agro-pecuária familiar: Um estudo de caso nas províncias de Benguela e do Cunene. https://www.adra-angola.org/artigos/estudo-sobre-a-partticipacao-da-mulher-na-producao-agro-pecuaria-familiar. Accessed 17 June 2025.

[78] Mama A. We will not be pacified’: from freedom fighters to feminists. Eur J Womens Stud. 2020;27(4):362–80. 10.1177/1350506820953459.

[79] Salo E, Mama A. Talking about feminism in Africa. Agenda: Empower Women Gend Equity. 2001;(50):58–63.

[80] Mohanty CT. Feminism without borders: decolonizing theory, practicing solidarity. Durham: Duke University Press; 2003.

[81] Hancock AM. When multiplication doesn’t equal quick addition: examining intersectionality as a research paradigm. Perspect Polit. 2007;5(1):63–79.

[82] Ngohengo JK. Modernism and the change of African gender relations: historical discourses. Afr J Hist Cult. 2021;13(2):110–5.

[83] Hankivsky O, Grace D, Hunting G, Giesbrecht M, Fridkin A, Rudrum S, et al. An intersectionality-based policy analysis framework: critical reflections on a methodology for advancing equity. Int J Equity Health. 2014;13(1):119–119. 25492385 10.1186/s12939-014-0119-x PMC4271465

[84] Bacchi CL. Analysing policy: what’s the problem represented to be? Frenchs Forest, N.S.W: Pearson; 2009.

[85] Hyder A, Syed S, Puvanachandra P, Bloom G, Sundaram S, Mahmood S, et al. Stakeholder analysis for health research: case studies from low- and middle-income countries. Public Health (London). 2010;124(3):159–66. 10.1016/j.puhe.2009.12.006 20227095

[86] United States Agency for International Development (USAID). The Demographic and Health Survey program. DHS model questionnaires 2014, adapted for Angola. https://dhsprogram.com/Methodology/Survey-Types/DHS-Questionnaires.cfm#CP_JUMP_16179. Accessed 21 Nov 2023.

[87] Coates J, Swindale A, Bilinsky P. Household food insecurity access scale (HFIAS) for measurement of food access: indicator guide: version 3. Washington, DC: FHI360/FANTA; 2007.

[88] World Health Organization (WHO). WHO multi-country study on women’s health and domestic violence against women: initial results on prevalence, health outcomes and women’s responses. 2005. https://iris.who.int/handle/10665/43309. Accessed 17 May 2025.

[89] Johansson M, Preuter M, Karlsson S, Möllerberg M-L, Svensson H, Melin J. Valid and reliable? Basic and expanded recommendations for psychometric reporting and quality assessment [Preprint]. 2023. 10.31219/osf.io/3htzc. Accessed 20 June 2023.

[90] Buchardt AS, Christensen KB, Jensen SN. Visualizing Rasch item fit using conditional item characteristic curves in R. Psychol Test Assess Model. 2023;65:206–19.

[91] Hagell P, Westergren A. Sample size and statistical conclusions from tests of fit to the Rasch model according to the Rasch unidimensional measurement model (Rumm) program in health outcome measurement. J Appl Meas. 2016;17(4):416–31. 28009589

[92] Christensen KB, Makransky G, Horton M. Critical values for yen’s *Q*_3_: identification of local dependence in the Rasch model using residual correlations. Appl Psychol Meas. 2017;41(3):178–94. 10.1177/0146621616677520. 29881087 10.1177/0146621616677520 PMC5978551

[93] Tutz G. On the structure of ordered latent trait models. J Math Psychol. 2020;96:102346. 10.1016/j.jmp.2020.102346.

[94] Komboz B, Strobl C, Zeileis A. Tree-based global model tests for polytomous Rasch models. Educ Psychol Meas. 2018;78(1):128–66. 10.1177/0013164416664394. 29795950 10.1177/0013164416664394 PMC5965621

[95] Bland JM, Altman DG. Statistical methods for assessing agreement between two methods of clinical measurement. Lancet. 1986;327(8476):307–. 10.1016/S0140-6736(86)90837-8. 10. 2868172

[96] Mallinckrodt B, Tekie YT. Item response theory analysis of working alliance inventory, revised response format, and new brief alliance inventory. Psychother Res. 2016;26(6):694–718. 10.1080/10503307.2015.1061718. 26549302 10.1080/10503307.2015.1061718

[97] R Core Team. R: A language and environment for statistical computing. Vienna, Austria: R Foundation for Statistical Computing. 2024. https://www.R-project.org/ . Accessed 17 June 2025.

[98] Mair P, Hatzinger R. Extended Rasch modeling: the eRm package for the application of IRT models in R. J Stat Softw. 2007;20. 10.18637/jss.v020.i09.

[99] Buchardt A, Jensen SN, Christensen KB. RASCHplot: Visualisation tool for validity of Rasch models. R package version 0.1.0. 2022 https://github.com/ERRTG/RASCHplot/ . Accessed 17 June 2025.

[100] Wilke CO. Cowplot: streamlined plot theme and plot annotations for’ggplot2’. CRAN: Contributed Packages. 2015.

[101] Pedersen TL. Patchwork: the composer of plots. CRAN: Contributed Packages. 2019.

[102] Adams RJ, Wu ML, Wilson M. The Rasch rating model and the disordered threshold controversy. Educ Psychol Meas. 2012;72(4):547–73.

[103] Andrich D. Controversy and the Rasch model: a characteristic of incompatible paradigms? Med Care. 2004;42(1):I–7. 10.1097/01.mlr.0000103528.48582.7c 14707751

[104] World Medical Association. World medical association declaration of helsinki: ethical principles for medical research involving human participants. JAMA. 2025;333(1):71–4. 39425955 10.1001/jama.2024.21972

