# Supplementary material for: Gender norms in sexual and reproductive health and rights: insights from young Angolan women and the development of a context-specific questionnaire (2021–2022)
Source: Arch Public Health. 2025 Dec 23;84:18. doi: 10.1186/s13690-025-01820-z (PMC12836931; doi:10.1186/s13690-025-01820-z)
Supplement: Supplementary file 1 — Supplementary Material 1. [file 13690_2025_1820_MOESM1_ESM.pdf]

Additional file 1. Overview of key informants working in Angola in the field of sexual and reproductive health and rights, and the interview guide used for qualitative data collection. All qualitative data was collected in Angola in 2021.

A: Overview of key informants' organisational characteristics and interview dates.

Organisational characteristic	Health and rights focus	Organisational reach	Date of interview
**Government department**			
National direction of public health	Community health	National	2021-07-26
National direction of public health	Adolescent health	National	2021-07-26
National direction of public health	Mental health	National	2021-07-27
National direction of public health	Gender equality	National	2021-07-29
National direction of public health	Gender equality	National	2021-07-29
Provincial direction of public health	Women's health	Provincial	2021-08-04
Provincial direction of public health	Sexual and reproductive health and rights	Municipal	2021-08-05
Provincial direction of public health	Maternal health	Municipal	2021-08-05
Provincial direction of public health	Maternal health	Municipal	2021-08-06
Provincial direction of public health	Sexual and reproductive health and rights	Municipal	2021-08-06
**National non-governmental organisation**			
Women's rights	Gender equality	Municipal	2021-08-04
Women's rights	Gender equality	National	2021-08-04
Community development	Gender equality	Provincial	2021-08-05
Community development	Gender equality	Provincial	2021-08-06
Women's rights	Gender equality	National	2021-08-06
Community development	Gender equality	Provincial	2021-08-09
Women's rights	Gender equality	National	2021-08-27
Women's rights	Gender equality	National	2021-09-03
**Non-Angolan government**			
International development agency	Maternal health	Provincial	2021-07-30
International development agency	Maternal health	National	2021-08-10
International development agency	Women's health and rights	National	2021-08-25
**International non-governmental organisation**			
Health systems strengthening	Gender equality	National	2021-07-27
Community development	Social determinants of health	Provincial	2021-08-03
Community development	Social determinants of health	Provincial	2021-08-04
Community development	Social determinants of health	Provincial	2021-09-16

B: Interview guide for qualitative key informant interviews involved in identifying relevant survey topics and sexual and reproductive health and rights themes (Angola, 2021).

General Perspectives on Women's Health and Rights

1. From your perspective:
   a. What are the main challenges currently facing women's health in Angola?
   b. What are the main challenges related to women's human rights in Angola?
2. In your opinion, what are the key barriers to improving:
   a. Women's health?
   b. Women's rights (e.g. the right to health, social security)?

Organisational Focus and Activities

3. How would you describe the main focus of your organisation?
4. What specific activities does your organisation undertake?
   (probing: target groups, geographical areas, health-related themes, etc.)
5. Could you elaborate on any programmes that focus specifically on women's health and rights?
   (probing: details such as target groups, geographic coverage, etc.)
6. Regarding these programmes:
   a. What are the main challenges in achieving the intended goals? *(probing: elaborate on why these challenges exist)*
   b. What would an ideal scenario look like for successful implementation of the programmes? *(probing: elaborate on why this would be ideal)*
7. What factors determine the areas your organisation prioritises?
   (probing: elaborate on how priorities and decisions are established; on what basis—e.g. mandate, funding sources, strategic focus)

Wider Programming and Collaboration

8. Are you aware of other organisations or programmes—national or international—working on similar themes?
9. Does your organisation collaborate with any of these actors?
   (probing: elaborate on the nature of collaborations)

Reflections on Gaps and Opportunities

10. In your view, what is needed to increase interest and engagement in women's health and rights?
11. Do you believe there are specific themes related to women's health and rights that are often overlooked, neglected, or considered taboo?
    (probing: elaborate on why they think this is the case)
