# Supplementary material for: Gender norms in sexual and reproductive health and rights: insights from young Angolan women and the development of a context-specific questionnaire (2021–2022)
Source: Arch Public Health. 2025 Dec 23;84:18. doi: 10.1186/s13690-025-01820-z (PMC12836931; doi:10.1186/s13690-025-01820-z)
Supplement: Supplementary file 2 — Supplementary Material 2. [file 13690_2025_1820_MOESM2_ESM.pdf]

Additional file 2. Overview of study variables and corresponding survey questions included in the larger SADIMA study questionnaire for the cross-sectional study with young women conducted in Angola in 2022.

Overview of study variables listed alphabetically, together with their original references, SADIMA survey items, and variable construction.

Variable name	Reference	SADIMA questionnaire			Category construct explanation
		Number	Question	Response options	
Acquired information about pregnancy and childbirth complications	1	Q84	Have you ever received information about health problems related to pregnancy or childbirth (what it is, what to do, where to get help)?	a) Yes<br>b) No	• Yes (1): if a on Q84<br>• No (0): if b on Q84
Area of residency	1	NA	Predefined	NA	• Urban (1): Luanda city centre and outskirts, urbanised areas of Huambo, Bailundo Saurimo municipalities<br>• Rural (2): rural areas (all other than those above)
Fertile period knowledge	2	Q94	Between one menstrual period and another, are there days when a woman is more likely to get pregnant?	a) Yes<br>b) No<br>a) Don't know	• Yes (1): if a on Q94 and b on Q95<br>• No (0): if b or c on Q94, or a on Q94 and a on Q95
		Q95	If yes, these days are…	Asked if a on Q94, Yes / No to each:<br>a) During menstruation<br>b) At the midpoint between menstrual periods	
Formal education	2	Q15	Have you ever attended school?	a) Yes<br>b) No	• High (2): if d or e on Q17<br>• Medium (1): if c on Q17<br>• Low (0): if b on Q15, or a or b on Q17
		Q17	What is the highest class you have completed?	Asked if a on Q15:<br>a) 1-4 class<br>b) 5-6 class<br>c) 7-9 class<br>d) 10-13 class<br>e) University studies	
Gender norms	People have different ideas about families and what is acceptable behaviour for men and women. We will now make some statements about relationships between men and women. We are not asking for your opinion, but for the views of people in this neighbourhood, village or community. Please indicate the option that you think is closest to the views of people in this neighbourhood, village or community.				
	People in this neighbourhood, in this village or commune think that… [repeated before each statement]				• SRHR-supportive norm: if 4 or 5<br>• Non-supportive norm: if 1-3
	3 (chap 5), 4	Q38	…is important for girls to study.	5 = TOTALLY AGREE; 4 = AGREE; 3 = DEPENDS (NEITHER AGREE OR DISAGREE); 2 = DISAGREE; 1 = TOTALLY DISAGREE	

	3 (chap 4, 6, 8), 4-8	Q39	…men should have more influence than women on important family and domestic decisions.	1 = TOTALLY AGREE; 2 = AGREE; 3 = DEPENDS (NEITHER AGREE OR DISAGREE); 4 = DISAGREE; 5 = TOTALLY DISAGREE	
	3 (chap 1), 5,6	Q40	…a woman has the right to say NO to sex with her partner for any reason.	5 = TOTALLY AGREE; 4 = AGREE; 3 = DEPENDS (NEITHER AGREE OR DISAGREE); 2 = DISAGREE; 1 = TOTALLY DISAGREE	
	3 (chap 5), 8	Q41	…a good woman shouldn't question her partner's opinions, even if she doesn't agree with him.	1 = TOTALLY AGREE; 2 = AGREE; 3 = DEPENDS (NEITHER AGREE OR DISAGREE); 4 = DISAGREE; 5 = TOTALLY DISAGREE	
	3 (chap 4), 8	Q42	…a real woman only after becoming a mother.	1 = TOTALLY AGREE; 2 = AGREE; 3 = DEPENDS (NEITHER AGREE OR DISAGREE); 4 = DISAGREE; 5 = TOTALLY DISAGREE	
	1, 4, 8	Q43	…it is shameful to talk about experiences of sexual abuse, even with close family and friends.	1 = TOTALLY AGREE; 2 = AGREE; 3 = DEPENDS (NEITHER AGREE OR DISAGREE); 4 = DISAGREE; 5 = TOTALLY DISAGREE	
	1, 5,6	Q44	…it is normal for girls to have children before the age of 18.	1 = TOTALLY AGREE; 2 = AGREE; 3 = DEPENDS (NEITHER AGREE OR DISAGREE); 4 = DISAGREE; 5 = TOTALLY DISAGREE	
	3 (chap 4, 6), 4	Q45	…a woman should tolerate violence from her partner/husband to keep the family together.	1 = TOTALLY AGREE; 2 = AGREE; 3 = DEPENDS (NEITHER AGREE OR DISAGREE); 4 = DISAGREE; 5 = TOTALLY DISAGREE	
	1, 8	Q46	…women should accept that men in positions of power (teachers, chiefs, police officers, etc.) demand sexual favours from them.	1 = TOTALLY AGREE; 2 = AGREE; 3 = DEPENDS (NEITHER AGREE OR DISAGREE); 4 = DISAGREE; 5 = TOTALLY DISAGREE	
	1, 4-8	Q47	…a couple should limit the number of pregnancies for the sake of the woman's health.	5 = TOTALLY AGREE; 4 = AGREE; 3 = DEPENDS (NEITHER AGREE OR DISAGREE); 2 = DISAGREE; 1 = TOTALLY DISAGREE	
	1	Q48	…a woman should be able to decide herself who to marry or have a close relationship with.	5 = TOTALLY AGREE; 4 = AGREE; 3 = DEPENDS (NEITHER AGREE OR DISAGREE); 2 = DISAGREE; 1 = TOTALLY DISAGREE	
Household night hunger	9	Q36	In the past four weeks, did you or any household member go to sleep at night hungry because there was not enough food?	a) Yes<br>b) No	• No (1): if b<br>• Yes (0): if a
Household wealth tertile	2	Q1-9	See below: Description of wealth index		
Intimate partner violence	2	Q24	What is your marital status? It is…	a) Married<br>b) Living maritally<br>c) Widow<br>d) Divorced/separated<br>e) In a relationship but not living together<br>f) Single<br>g) Never in a relationship	• No (1): if 0 on 120, 121, 122 and 123 (or NA = if never in a relationship (-88), missing (-99) or do not want to answer (-77))<br>• Yes (0): if a on one of 120, 121, 122 or 123

	10	Q120	Has your partner, boyfriend or date ever insulted, humiliated or threatened you?	Asked if a-f on Q24:<br>a) Yes<br>b) No	
		Q121	Has your partner, boyfriend or date ever slapped you, hit you or pushed you?	Asked if a-f on Q24:<br>a) Yes<br>b) No	
		Q122	Has your partner, boyfriend or date ever physically forced (forced) you to have sex when you didn't want to?	Asked if a-f on Q24:<br>a) Yes<br>b) No	
		Q123	Have you ever had sex when you didn't want to, for fear of the reaction of your partner, boyfriend or date?	Asked if a-f on Q24:<br>a) Yes<br>b) No	
Literate	2	Q15	Have you ever attended school?	a) Yes<br>b) No	• Yes (1): if a on Q15 and a on Q18<br>• No (0): if b on Q15 or b-d on Q18 (if Never attended school, not asked Q18, but included in No, i.e., illiterate)
		Q18	Now I would like you to read this sentence to me:<br>MOTHERS LOVE THEIR CHILDREN.	Asked if a on Q15:<br>a) Can read the whole sentence<br>b) Can read part of the sentence<br>c) Cannot read<br>d) Blind / visually impaired	
Menstrual autonomy	1	Q93	During your period, in which of the following situations do you feel that you can live like normal? (ANSWER EACH ONE)	a) Be with your partner (Yes/No)<br>b) Participate in domestic activities (Yes/No)<br>c) Continue to study or work (outside home) (Yes/No)<br>d) Spend time away (shopping, visiting friends etc) (Yes/No)	• Yes (1): if yes on c or d<br>• No (0): if no on c and d
Modern family planning awareness	2	Q98	Which of the following methods for delaying or avoiding pregnancy do you KNOW OF? (ANSWER EACH ONE)	e) Plants<br>f) Calendar method (periodic abstinence)<br>g) Interrupted coitus (sex)<br>h) Condom (camisola)<br>i) Pills<br>j) Injections and implants<br>k) Other	• Yes (1): if yes on Q98 d, e or f<br>• No (0): if no on Q98 d, e and f
Modern family planning use	2	Q99	Have you or your partner ever used a method to avoid or delay pregnancy?	a) Yes<br>b) No	• Yes, one type (1): if yes on Q100 d, e or f<br>• No (0): if no on Q100 d, e and f
		Q100	If yes, which method have you used? (ANSWER EACH ONE)	a) Plants<br>b) Calendar method (periodic abstinence)<br>c) Interrupted coitus (sex)<br>d) Condom (camisola)<br>e) Pills<br>f) Injections and implants<br>g) Other	

Pregnancy	2	Q67	Have you ever been pregnant?	a) Yes<br>b) No	• No (2) = if b on Q67<br>• Yes, 1st age ≥18 (1) = if a on Q67 and ≥18 on Q68<br>• Yes, 1st before age 18 (0) = if a on Q67 and >18 on Q68
		Q68	How old were you when you got pregnant for the first time?	Continuous	
Province	1	NA	Predefined	NA	• Luanda (1)<br>• Huambo (2)<br>• Lunda Sul (3)
Work	2	Q21	Have you had a job or business in the last 12 months?	a) Yes<br>No	• Yes, permanent (2) = If 1 on Q022<br>• Yes, temporary (1) = If 2 on Q022<br>• No (0) = If 0 on Q021
		Q22	Was this job or business permanent or temporary? (SELECT ONE)	a) Permanent (throughout the year)<br>Temporary (from time to time)	

Adaptation and development of the SADIMA wealth index from the original model

This study uses a wealth index that was developed for the SADIMA research project, further described in the methods section of the article. The wealth index is based on the Angola 2014 USAID household survey's area-type (urban/rural) adjusted wealth index [11]. The USAID wealth index was created using 12 survey questions, which are listed in Table B, below. The table also shows how these questions were adapted for the SADIMA wealth index survey. The USAID questions and response options were adapted for context and study population in two steps. Firstly, response options that received very low response rates in the Angola USAID household survey were merged or removed, in the latter case to be captured in the response option "Other" (see the term "relevance" in Table B comments). Secondly, as part of the development work for the full SADIMA questionnaire, the wording of questions and response options were adapted in accordance with feedback during the questionnaire validation work. This was done to ensure appropriateness, comprehensibility and sensitivity (see the term "comprehensibility" in Table B comments).

The creation of the SADIMA wealth index followed the principles described by the USAID project [12]. For each item category (NO.1-9 in the SADIMA questionnaire, see Table B) a dummy variable was created, i.e., all item answers were dichotomized to form a set of binary variables, say X, to give rise to the index. Since original binary variables may be somewhat redundant, a data reduction procedure was applied to X – either Principal Components Analysis (PCA) or Logistic Principal Components Analysis (LPCA). Either way the first component – $PCA_{(1)}$ or $LPCA_{(1)}$ – was used as the wealth index. The same procedure was repeated for five settings, namely only urban households, only semi-urban households, urban and semi-urban households, only rural households, and all households (overall). For the latter, the item "How many animals does your household own?" (USAID NO.

118, SADIMA NO. 6) was excluded. All analyses were performed using R Statistical Software (v4.3.1; R Core Team 2023) [13] more specifically the function *prcomp* and the package *logisticPCA* (v0.2) [14], respectively, for PCA and LPCA. Since the resulting sets PCA$_{(1)}$ and LPCA$_{(1)}$ were remarkably similar, either method of data reduction is applicable. For the SADIMA study's wealth index, the result from PCA is regarded in all data analysis. The correlation between the sets of weights (or loadings) from LPCA$_{(1)}$ and PCA$_{(1)}$ was equal to 0.71 whilst it was equal to 0.99 when comparing the respective household scores. Moreover the table below summarizes the correlation pattern (selected) between the sets of weights (or loadings) from PCA(1) and those relative to the Angola USAID Household survey 2015-2016 [15]. In the latter only modes Overall, Urban & Semi-urban, and Rural were implemented.

Correlation patterns between the SADIMA wealth index and the Angola USAID wealth index

**SADIMA**	**Angola USAID**		
	**Overall**	**Urban & Semi-urban**	**Rural**
Overall	0,81		
Urban		0,73	
Semi-urban		0,85	
Urban & Semi-urban		- 0,85	
Rural			0,66

Adaptation of SADIMA survey questions from the USAID Angola household survey 2014 wealth index

<table>
<tr><th colspan="3">Angola USAID questionnaire</th><th rowspan="2">Comment on adaptation</th><th colspan="3">SADIMA questionnaire</th></tr>
<tr><th>NO</th><th>Question</th><th>Response options</th><th>NO</th><th>Question</th><th>Response options</th></tr>
<tr><td>101</td><td>What is the main source of drinking water for the members of your household?</td><td rowspan="2">Piped water
11. Piped into dwelling
12. Piped to yard/plot
13. Piped to neighbor
14. Public tap/standpipe

21. Tube well or borehole
Dug well
31. Protected well
32. Unprotected spring
Water from spring
41. Protected spring
42. Unprotected spring</td><td rowspan="2">The USAID wealth index includes only question 101, but an amalgamation of the USAID 101 and 102 questions was used (the primary use of water, quantity wise, is not for drinking).

The technical answer options were popularized and combined for comprehensibility and relevance.</td><td rowspan="2">Q1</td><td rowspan="2">What is the main source of water supply used by the members of this household (for drinking, cooking, washing, bathing)?</td><td rowspan="2">a) Tap connected to public mains in your home (piped water/tank/well)
b) Tap connected to public mains at neighbor's house
c) Public tap / fountain
d) Tanker / van/ motorbike
e) Protected well / cistern
f) Unprotected well / spring / pond / river / canal
g) Other</td></tr>
<tr><td>102</td><td>What is the main source of water used by your household for other purposes such as cocking and handwashing?</td></tr>
</table>

		51. Rainwater<br>61. Tanker truck<br>71. Cart with small tank<br>81. Surface water (river/dam/lake/pond/stream/canal/irrigation channel)<br>91. Bottled water<br>96. Other (specify)				
109	What kind of toilet facility do members of your household usually use?	**Flush or pour flush toilet**<br>11. Flush to piped sewer system<br>12. Flush to septic tank<br>13. Flush to pit latrine<br>14. Flush to somewhere else<br>15. Flush, don't know here<br>**Pit latrine**<br>21. Ventilated improved pit latrine<br>22. Pit latrine with slab<br>23. Pit latrine without slab/open pit<br><br>31. Composting toilet<br>41. Bucket toilet<br>51. Hanging toilet/hanging latrine<br>61. No facility/bush/field<br>96. Other (pseicify)	Adapted:<br>The USAID wealth index includes only question 109, but an amalgamation of the USAID 109 and 112 questions was used for comprehensibility and relevance.	Q2	What type of sanitation facility do the members of this household use?	a) Inside the house (toilet / latrine / septic tank)<br>b) Outside the house but inside the yard (toilet / latrine / septic tank)<br>c) Outside the yard with some mechanism<br>d) No sanitation facility / outdoor / bush
112	Where is this toilet facility located?	1. In own dwelling<br>2. In own yard/plot<br>3. Elsewhere				
113	What type of fuel does your household mainly use for cooking?	01. Electricity<br>02. LPG<br>03. Natural gas<br>04. Biogas<br>05. Kerosene<br>06. Coal, lignite<br>07. Charcoal<br>08. Wood<br>09. Straw/shrubs/grass<br>10. Agricultural crop<br>11. Animal dung<br>95. No food cooked in household<br>96. Other (specify)	Adapted:<br>The technical answer options were popularized and combined for comprehensibility and relevance.	Q3	What is the main source of energy or fuel that your household uses for cooking?	a) Electricity<br>b) Natural gas<br>c) Coal<br>d) Firewood<br>e) Other
143	Observe main material of the roof of the dwelling. Record observation.	**Natural roofing**<br>11. No roof<br>12. Dung<br>**Rudimentary roofing**<br>21. rustic mat<br>22. Palm/bamboo<br>23. Wood planks<br>24. Cardboard	Adapted:<br>The technical answer options were popularized and combined for comprehensibility and relevance.	Q4	What kind of roof does the house where you live have?	a) Grass / Palm leaf / wood<br>b) Zinc sheets<br>c) Concrete slab / tile / lucalite / asbestos cement<br>d) Other

		**Finished roofing**<br>31. Metal<br>32. Wood<br>33. Calamine/Cement fiber<br>34. Ceramic tiles<br>35. Cement<br>96. Other (specify)				
144	Observe main material of the exterior walls of the dwelling. Record observation.	**Natural walls**<br>11. No walls<br>12. Cane/palm/trunks<br>13. Dirt<br>**Rudimentary walls**<br>21. Bamboo with mud<br>22. Stone with mud<br>23. Uncovered adobe<br>24. Plywood<br>25. Cardboard<br>26. Reused wood<br>**Finished walls**<br>31. Cement<br>32. Stone with lime/cement<br>33. Bricks<br>34. Cement blocks<br>35. Covered adobe<br>36. Wood planks/shingles<br>96. Other (specify)	Adapted:<br>The technical answer options were popularized and combined for comprehensibility and relevance.	Q5	What kind of walls does the house you live in have?	a) Zinc sheets / wood<br>b) Clay / pau-a-pique<br>c) Adobe<br>d) Cement / bricks / stone<br>e) Other
118	How many of the following animals does this household own?	a) Milk cows or bulls?<br>b) Other cattle?<br>c) Horses, donkeys, or mules?<br>d) Goats?<br>Sheep?<br>e) Chicken or other poultry?	Adapted:<br>The answer options were slightly adapted for comprehensibility.	Q6	How many animals does your household own?	Number of:<br>a) Dairy cows, ox, bulls or other type of livestock<br>b) Horses, donkeys or mules<br>c) Goats or sheep<br>d) Chickens or other birds<br>e) Other animals
119	Does any member of this household own any agricultural land?	1. Yes<br>2. No	As in the original instrument	Q7	Does your household own land (land for cultivation)?	a) Yes<br>b) No
121	Does your household have:	a) Electricity<br>b) A radio?<br>c) A television?<br>d) A non-mobile phone?<br>e) A computer?<br>f) A refrigerator?	Adapted:<br>The answer options Electricity and Non-mobile phone were removed, the former because "refrigerator" is a proxy for this, the latter because of rareness in this context.	Q8	Does your household own?	Yes / No to each:<br>a) Radio / music device<br>b) TV<br>c) Computer<br>d) Refrigerator
122	Does any member of this household own:	a) A watch?<br>b) A mobile phone?<br>c) A bicycle?<br>d) A motorcycle or motor scooter?<br>e) An animal-drawn cart?<br>f) A car or truck?	Adapted:<br>The answer options “watch”, “animal-drawn cart” and “boat” were removed, because of rareness in this context.	Q9	Does your household own?	Yes / No to each:<br>a) Bike<br>b) Motorbike<br>c) Car<br>d) Mobile phone

		g) A boat with a motor?				
116	How many rooms in this household are used for sleeping?	Rooms (number)	Not included in the SADIMA wealth index: The question is, in the USAID index, calculated in relation to the USAID household survey's information on all residents in the household. The question was excluded because of manageability and relevance, especially as households in rural areas often consist of several smaller dwellings.			
120	How many hectares of agricultural land do members of this household own?	Hectares (number)	Not included in the SADIMA wealth index: The question was excluded because of its technical nature that most likely would not meet the language use of the study population.			
123	Does any member of this household have a bank account?	1. Yes<br>2. No	Not included in the SADIMA wealth index: The question was excluded because the study population would not, in many cases, be able to answer it with sufficient certainty.			

References:

1. SADIMA: Survey question constructed specifically for the SADIMA study, often inspired by the USAID 2014.

2. USAID. *The Demographic and Health Survey program, DHS model questionnaires 2014, adapted for Angola*, Available at: https://dhsprogram.com/Methodology/Survey-Types/DHS-Questionnaires.cfm#CP_JUMP_16179.

3. Nanda, G. (2011) *Compendium of Gender Scales*. Washington, DC: FHI 360/C-Change.

4. Mosaiko, Research report on inclusive public policies from a gender perspective (in Portuguese: Mosaiko Relatório da pesquisa sobre políticas públicas inclusivas numa perspectiva de género, 2019-2021), Luanda: Tipografia Coimbra. 2021. Available from: https://mosaiko.op.org/wp-content/uploads/2019/05/PAPPIA-Relatorio-de-Pesquisa-Web.pdf.

5. Mouzinho Â, Cutaia S. Reflections on feminist organising in Angola. Feminist Africa. 2017 Dec 1(22):33-51.

6. Mwana Pwo Association. Just a Child: Exploring Child Marriage in Angola. 2021. (in Portuguese: Associação Mwana Pwo. Apenas Uma Criança. Explorando Casamento Infantil em Angola).

7. Action for Rural and Environmental Development (ADRA), The Participation of Women in Family Agricultural and Livestock Production, A Case Study in the Provinces of Benguela and Cunene. 2020. (in Portuguese: Acção para o Desenvolvimento Rural e Ambiende (ADRA), A Participação da Mulher na Produção Agro-Pecuária Familiar, Um Estudo de Caso nas Províncias de Benguela e do Cunene.)

8. Telo FCA. Reproductive rights in Angola: Does the utopia of rights or the right to utopia? (in Portuguese: Direitos reprodutivos em Angola: A utopia does direitos ou o direito à utopia?), in: Ferreira LFG, de Moura LLD, Franca MHO, Araújo MMB. Annals: IX International Seminar on Human Rights of UFPB. Challenges and

Perspectives of Democracy in Latin America (in Portuguese: Anais: IX Seminário Internacional de Direitos Humanos da UFPB. Desafios e perspectivas da Democracia na América Latina). 2017. Available from: http://www.cchla.ufpb.br/ncdh/wp-content/uploads/2017/09/IX-SIDH_Anais-Eletr%C3%B4nicos-2.pdf

9. Coates J, Swindale A, Bilinsky P. (2007) *Household Food Insecurity Access Scale (HFIAS) for Measurement of Food Access: Indicator Guide: Version 3* (Washington, USA: USAID).

10. World Health Organization (WHO). *WHO multi-country study on women's health and domestic violence against women : initial results on prevalence, health outcomes and women's responses* / authors: Claudia Garcia-Moreno ... [et al.]. World Health Organization. 2005. Available at: https://iris.who.int/handle/10665/43309.

11. "Wealth index construction; Wealth index; Topics; The USAID program," 2021, accessed 2021-07-25, 2021, https://USAIDprogram.com/methodology/survey/survey-display-477.cfm.

12. Rutsein, SO, Johnson, K, *The USAID wealth index. USAID comparative reports No. 6.*, USAID, Calverton, Maryland, USA: Macro, O, 2004); Rutstein, SO, *The USAID wealth index: Approaches for rural and uban aeas*, Demographic and health research division, USAID, Calverton, USA: Inc., MI, 2008); Croft, TN et al., *Guide to USAID Statistics. Demographic and health surveys methodology*, USAID, Rockville, USA: ICF, 2023).

13. "R: A language and environment for statistical computing," R Foundation for Statistical Computing,, 2023, https://www.R-project.org/.

14. Landgraf, AJ, Lee, Y, *Dimensionality Reduction for Binary Data through the Projection of Natural Parameters*, The Ohio State University, 2015).

15. Instituto Nacional de Estatística et al., *Inquérito de indicadores múltiplos e de saúde em Angola 2015–2016*, Instituto Nacional de Estatística (INE), The International Coaching Federation Maryland Chapter (ICF-MD), Luanda, Angola, e Rockville, Maryland USA, 2017).
