# Supplementary material for: Gender norms in sexual and reproductive health and rights: insights from young Angolan women and the development of a context-specific questionnaire (2021–2022)
Source: Arch Public Health. 2025 Dec 23;84:18. doi: 10.1186/s13690-025-01820-z (PMC12836931; doi:10.1186/s13690-025-01820-z)

Additional file 3. Flow chart illustrating the inclusion and exclusion of study participants in the cross-sectional study of young women conducted in Angola 2022, presented by province based on completed of gender norms questionnaire items.

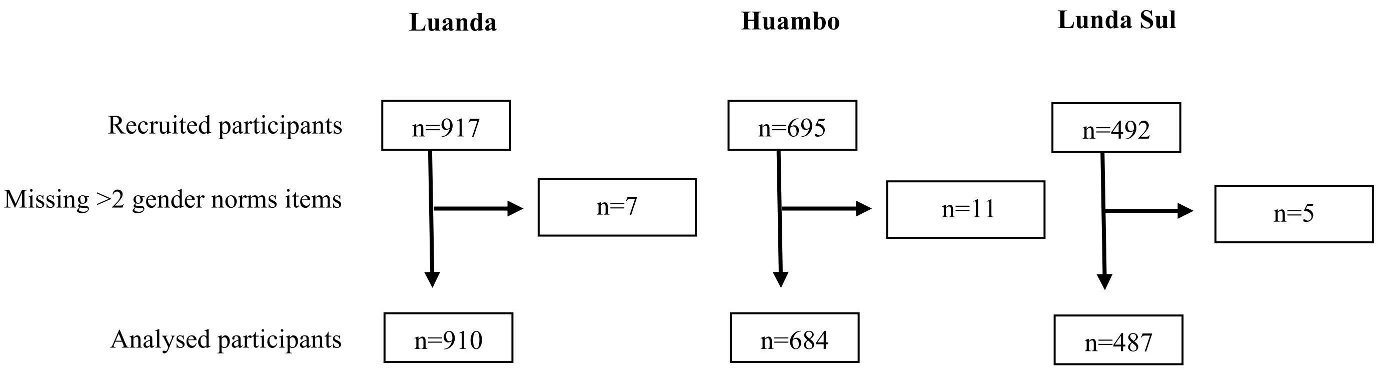

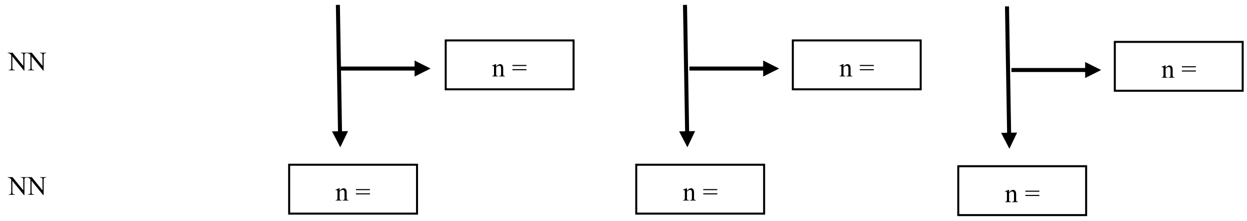

Supplement: Supplementary file 3 — Supplementary Material 3. [file 13690_2025_1820_MOESM3_ESM.pdf]
