# Supplementary material for: Gender norms in sexual and reproductive health and rights: insights from young Angolan women and the development of a context-specific questionnaire (2021–2022)
Source: Arch Public Health. 2025 Dec 23;84:18. doi: 10.1186/s13690-025-01820-z (PMC12836931; doi:10.1186/s13690-025-01820-z)
Supplement: Supplementary file 4 — Supplementary Material 4. [file 13690_2025_1820_MOESM4_ESM.pdf]

Additional file 4 Rasch analysis

to *Gender norms in sexual and reproductive health and rights: Insights from young Angolan women and the development of a context-specific questionnaire (2021-2022)* by Priebe et al

This file collects results from the Rasch analysis of the questionnaire based on the data from the cross-sectional study with young women in Angola in 2022.

Table A Overview of results from the Rasch analysis 2
Table B Outfit and infit statistics 6

Figure A Results of the local dependence analysis 3
Figure B Item characteristic curves for the original 5-point Likert scale. 4
Figure C Item characteristic curves for the 4-point Likert scale 5
Figure D CICC plots based on the 5-point Likert scale 7
Figure E CICC plots based on the 4-point Likert scale 8
Figure F Partial credit tree 9
Figure G DIF plot by province. 10
Figure H Comparison of item threshold locations 11
Figure I Bland-Altman plot for estimated persons' locations based a model common for all three provinces and on a model accounting for DIF by province 12
Figure J Histograms of persons' locations in the three different provinces 12
Figure K Item hierarchy 13

Table A Overview of results from the Rasch analysis applied to the original 5-point Likert scale and to the modified 4-point Likert scale (merged middle category), the latter both for the whole dataset and stratified by province. Data from the cross-sectional study with young women in Angola in 2022.

		**Original scale**<br><br>**N=2801**	**Merged middle category**	**Merged middle category, Luanda**	**Merged middle category, Huambo**	**Merged middle category, Lunda Sul**
**Unidimensionality**	Max. eigenvalue	1.5	1.38	1.47	1.46	1.47
	% explained variance (1st PC)	14.3	13.6	14.6	14.3	14.5
**Local dependence**	Q3-mean(Q3),<br>shown is the highest value, local dependence is indicated if >0.2	0.12 (Maternal health x Marital consent)	0.11 (Decision equality x Freedom of expression; Marital consent x Maternal health)	0.2 (Freedom from violence x Bodily autonomy)	0.13 (Decision equality x Freedom of expression; Freedom from violence x Freedom of expression	0.17 (Maternal health x Marital consent)
**Ordering of categories**		Middle category disordered for all items	Ordered	ordered	ordered	ordered
**Item fit**	Misfit visual– overfitting (underlined if flagged by infit/outfit)	Freedom from violence<br>Identity diversity<br>Bodily autonomy	Freedom from violence<br>Identity diversity<br>Bodily autonomy	Freedom from violence<br>Bodily autonomy	Freedom from violence<br>Bodily autonomy	Freedom from violence
	Misfit visual – underfitting (underlined if flagged by infit/outfit)	Marital consent<br>Decision equality	Marital consent	Marital consent<br>Decision equality	Marital consent	Marital consent
**Targeting**		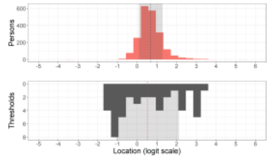	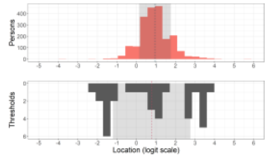	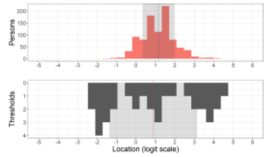	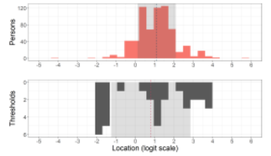	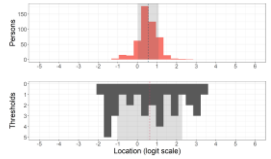
**Hierarchy**	Lowest to highest	1. Education access<br>2. Bodily autonomy<br>3. Relationship autonomy<br>...<br>9. Identity diversity<br>10. Marital consent<br>11. Decision equality	1. Education access<br>2. Bodily autonomy<br>3. Relationship autonomy<br>...<br>9. Identity diversity<br>10. Marital consent<br>11. Decision equality	1. Education access<br>2. Bodily autonomy<br>3. Relationship autonomy<br>...<br>9. Non-stigmatisation<br>10. Marital consent<br>11. Decision equality	1. Education access<br>2. Bodily autonomy<br>3. Relationship autonomy<br>...<br>9. Freedom of expression<br>10. Marital consent<br>11. Decision equality	1. Education access<br>2. Bodily autonomy<br>3. Relationship autonomy<br>...<br>9. Reproductive maturity<br>10. Decision equality<br>11. Identity diversity
**Reliability**	TIF	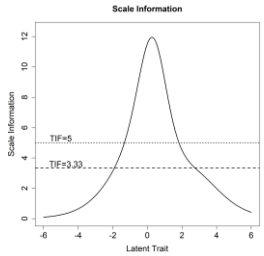	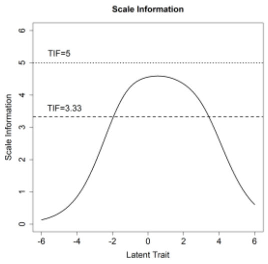	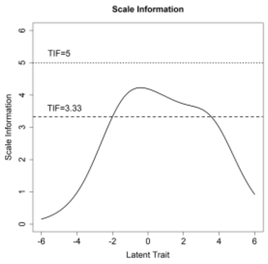	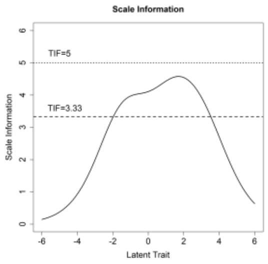	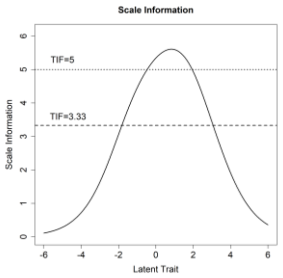
	full responses with TIF>3.3	98.6%	98.6%	99.2%	97.1%	100%

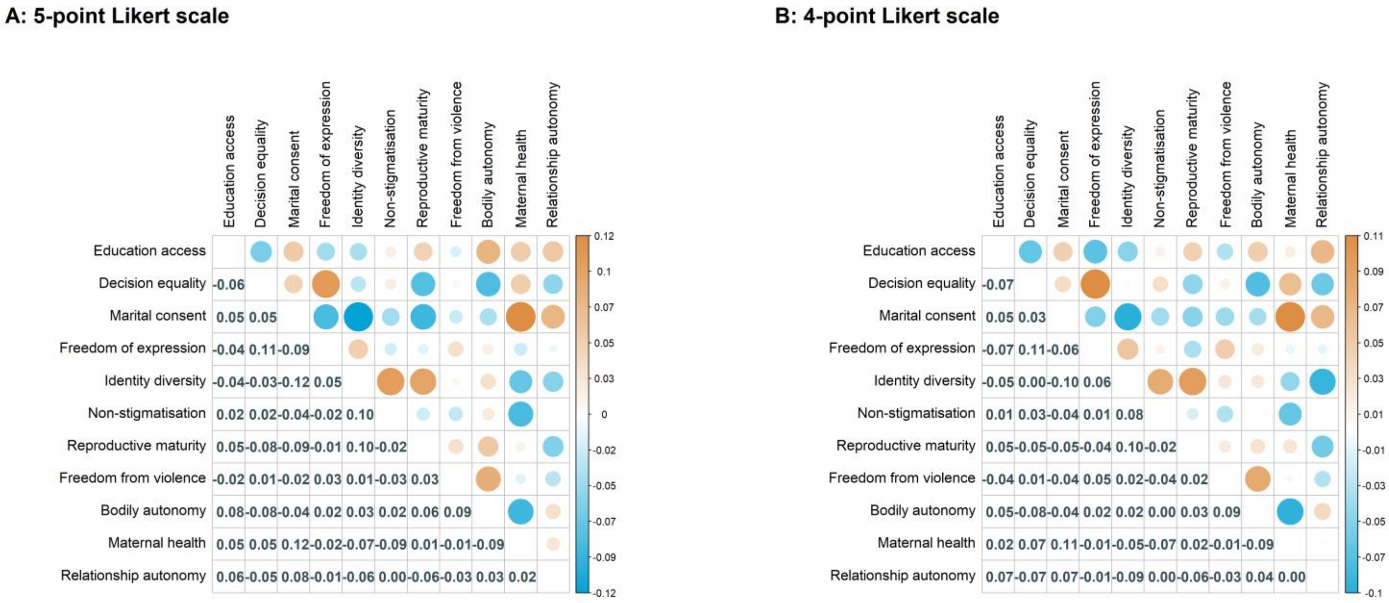

Figure A Results of the local dependence analysis. Shown is the exceedance of Yen's Q3 statistics over the mean value of these statistics in the data set. Values above 0.2 are considered indicative of local dependence. Data from the cross-sectional study with young women in Angola in 2022.

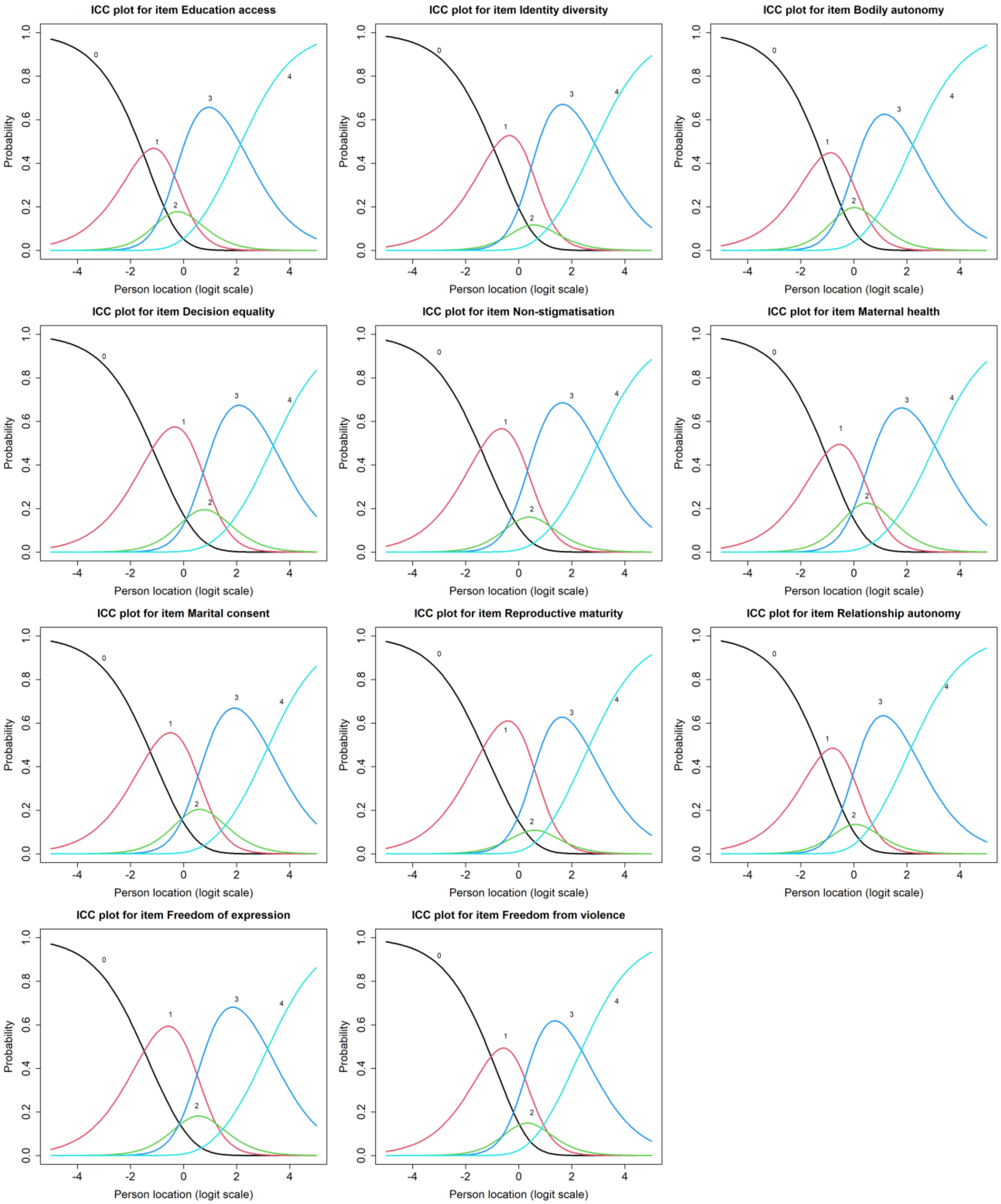

Figure B Item characteristic curves (ICC) for the original 5 response options on the 5-point Likert scale. The middle response option (labelled as 2) is always disordered. Data from the cross-sectional study with young women in Angola in 2022.

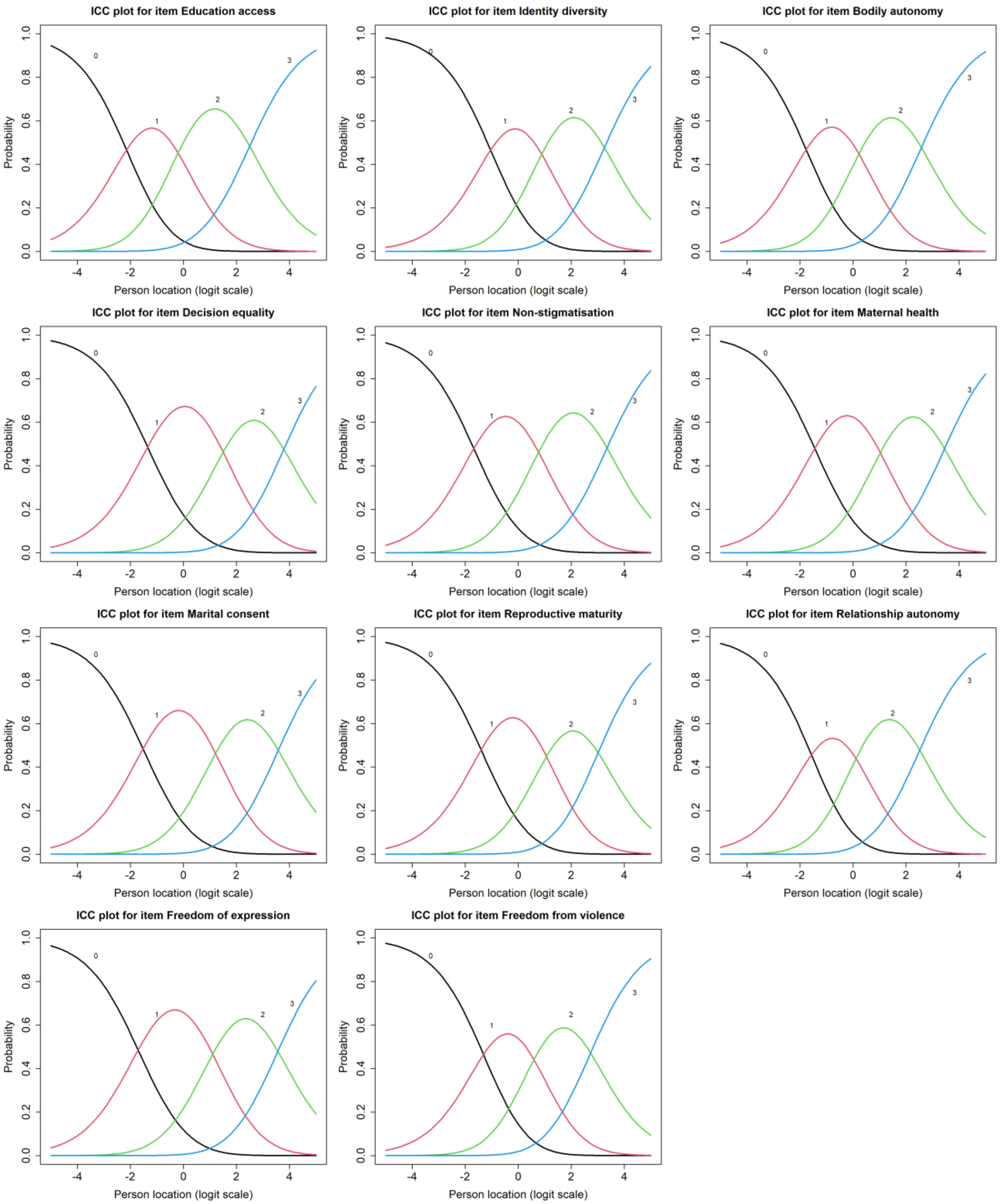

Figure C Item characteristic curves (ICC) after collapsing the 5-point Likert scale into a 4-point one. All items show ordered response categories. Data from the cross-sectional study with young women in Angola in 2022.

Table B Outfit and infit statistics for the 5-point Likert scale (right) and the modified 4-point Likert scale (left). Statistics indicating overfit (<-2 in normalized infit and outfit, <0.7 in mean square infit and outfit) and underfit (>2 in normalized infit and outfit, or >1.3 in mean square infit and outfit) are in bold. Data from the cross-sectional study with young women in Angola in 2022.

	**5-point Likert scale**				**4-point Likert scale**			
Outfit and infit statistics based on 100 subsamples of size 350	**Mean square outfit**	**Mean square infit**	**z-outfit**	**z-infit**	**Mean square outfit**	**Mean square infit**	**z-outfit**	**z-infit**
**Education access**	0.91	0.92	-0.95	-0.99	0.95	0.95	-0.71	-0.67
**Decision equality**	1.08	1.05	1.27	0.82	1.02	1.01	0.23	0.14
**Marital consent**	1.19	1.13	**2.80**	**2.01**	1.08	1.06	1.00	0.84
**Freedom of expression**	0.94	0.91	-0.95	-1.48	0.89	0.89	-1.50	-1.56
**Identity diversity**	0.88	0.86	-1.83	**-2.39**	0.84	0.83	**-2.38**	**-2.51**
**Non-stigmatisation**	1.05	1.02	0.68	0.34	1.00	1.00	0.07	-0.01
**Reproductive maturity**	0.92	0.90	-1.31	-1.70	0.88	0.87	-1.78	-1.90
**Freedom from violence**	0.78	0.78	**-3.38**	**-3.63**	0.77	0.76	**-3.42**	**-3.62**
**Bodily autonomy**	0.81	0.85	**-2.49**	**-2.10**	0.81	0.82	**-2.68**	**-2.57**
**Maternal health**	0.97	0.95	-0.45	-0.85	0.92	0.91	-1.18	-1.29
**Relationship autonomy**	1.00	1.00	0.06	0.03	0.97	0.98	-0.34	-0.29

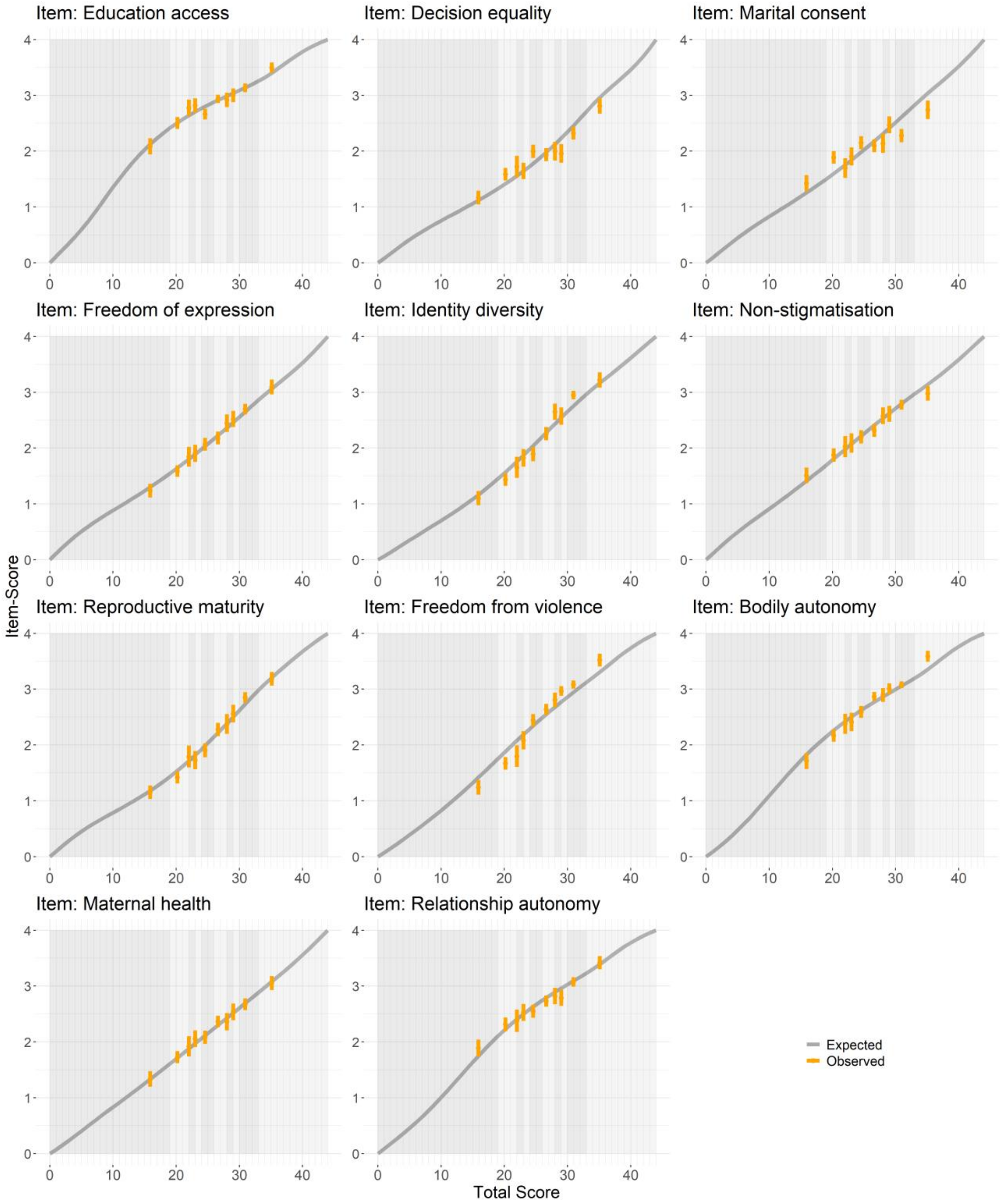

Figure D Conditional item characteristic curve (CICC) plots based on the 5-point Likert scale contrasting the model predicted mean score (expected) with the observed average score conditional on the total score. The shading indicates the range of total scores of the participants who were involved in the calculation of the average observed score on that item. Data from the cross-sectional study with young women in Angola in 2022.

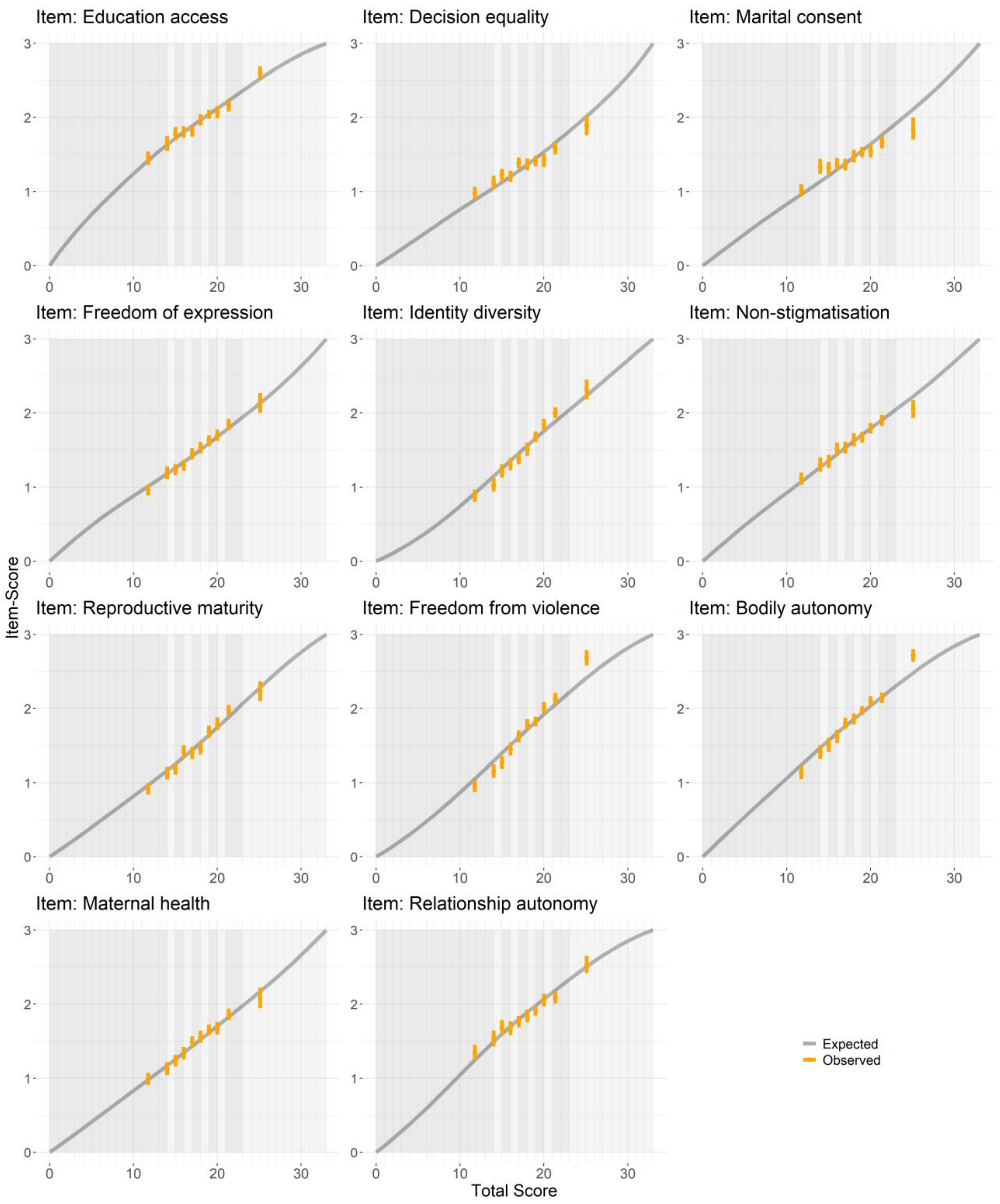

Figure E Conditional item characteristic curve (CICC) plots based on the 4-point Likert scale contrasting the model predicted mean score (expected) with the observed average score conditional on the total score. The shading indicates the range of total scores of the participants who were involved in the calculation of the average observed score on that item. Data from the cross-sectional study with young women in Angola in 2022.

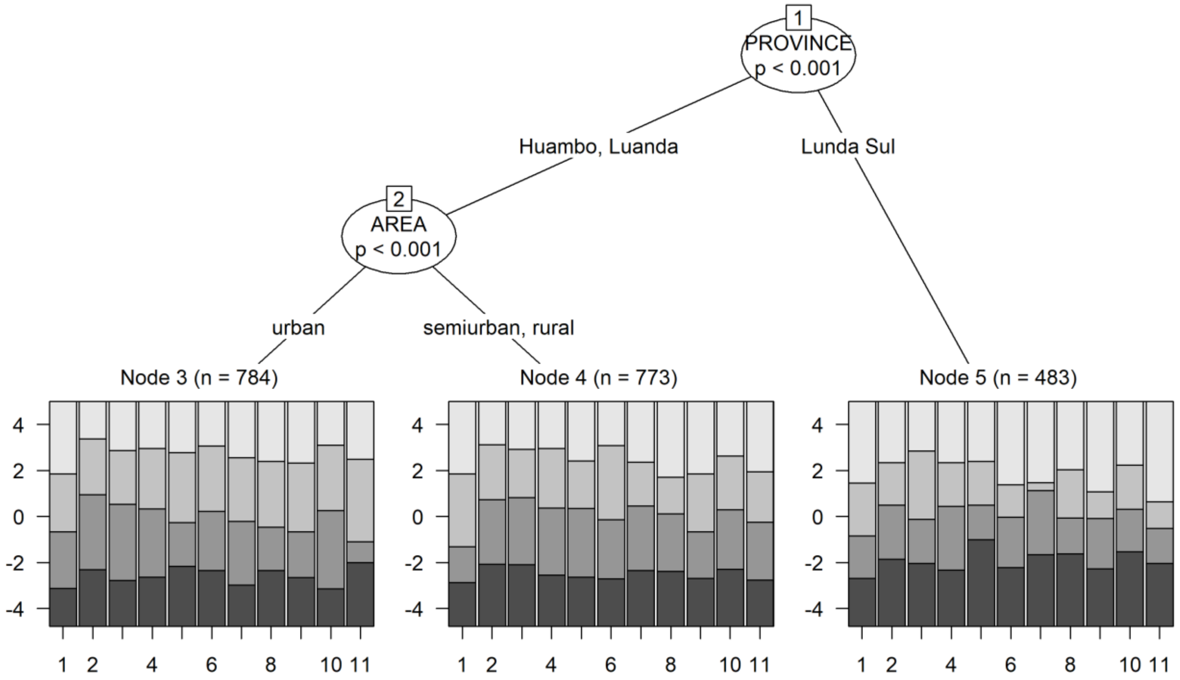

Figure F Partial credit tree based on 2040 participants, who have full information on the splitting characteristics: province, household wealth, literacy and living area type. The strongest difference in functioning was identified between provinces, where Lunda Sul seems to be markedly different from the other two. In the next step, Huambo and Luanda participants are split according to the area type. However, this split is very close to a split along the provinces themselves, since 0% of Huambo participants and 88% of Luanda participants are living in what is classified as urban areas. Province is also providing the second-best split in node 2. That is why we further examined differential item functioning (DIF) according to provinces. the cross-sectional study with young women in Angola in 2022.

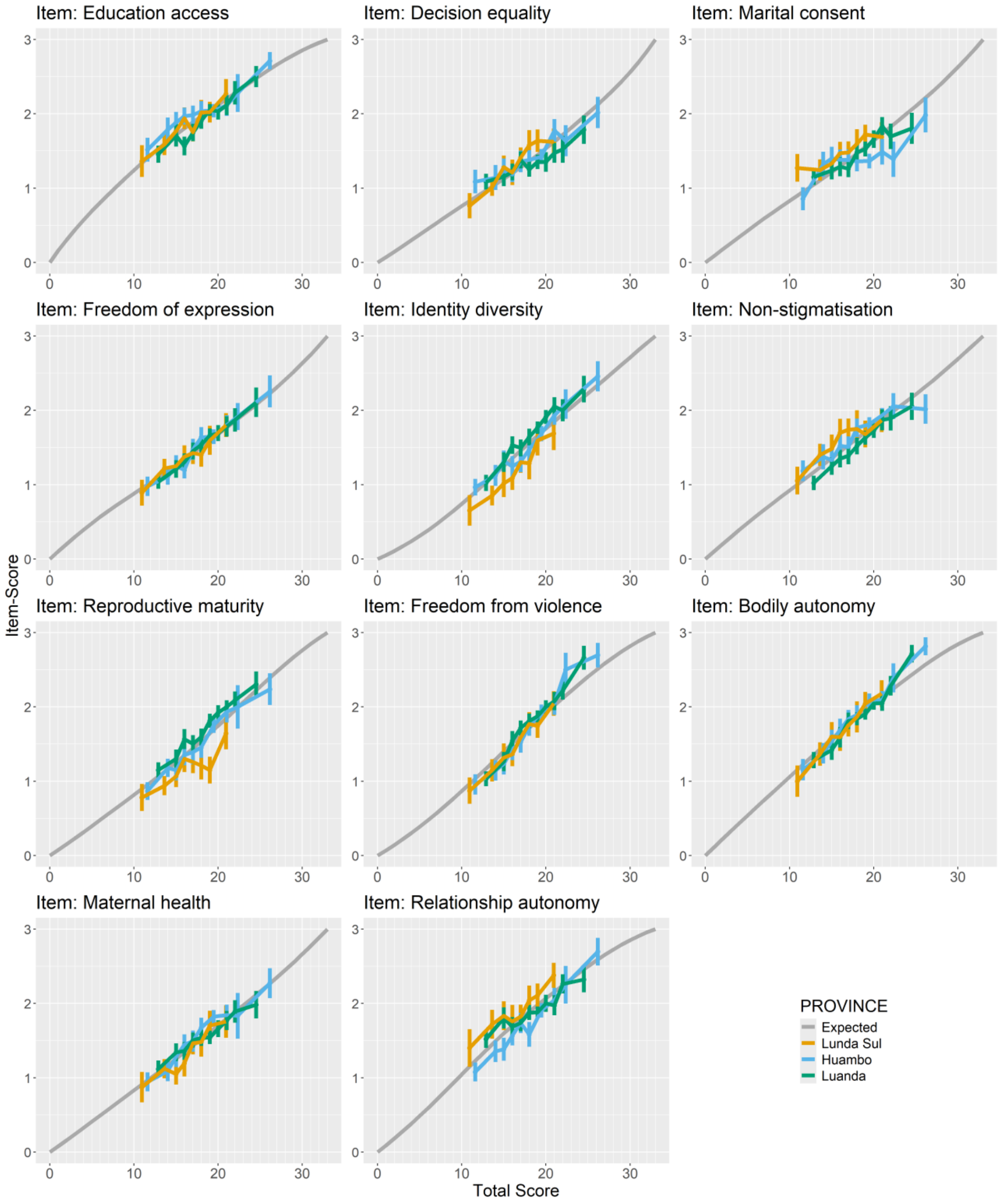

Figure G Differential item functioning *(*DIF) plot by province. Conditional on total score, the observed average scores on an item within each province are contrasted with the mean score predicted by the model that does not account for DIF. Data from the cross-sectional study with young women in Angola in 2022.

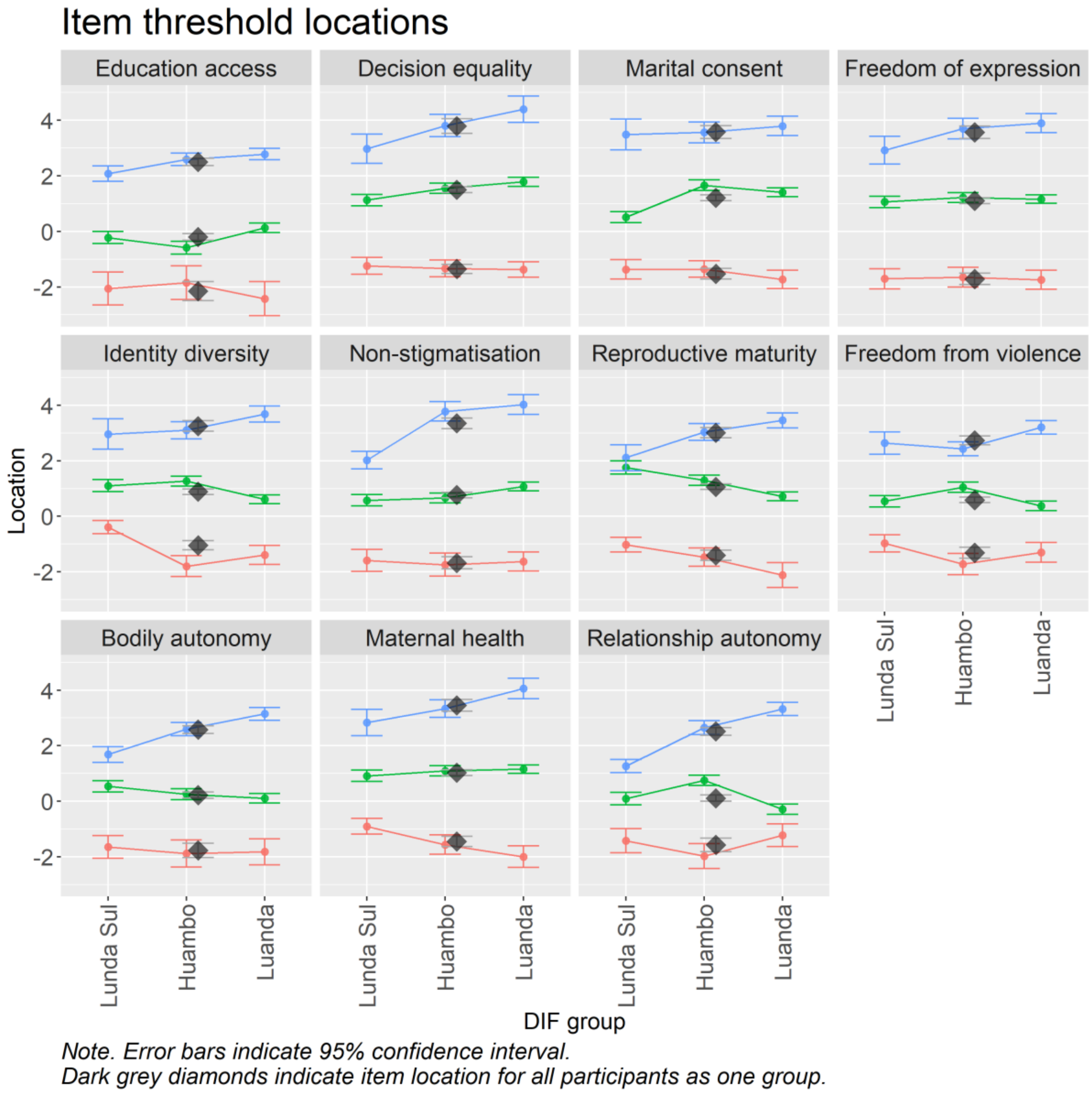

Figure H Comparison of item threshold locations (threshold T1 red, threshold T2 green and threshold T3 blue) as estimated in the provinces. For Freedom of expression the lines are almost parallel; thus, this item seems to function very similar across the provinces. Data from the cross-sectional study with young women in Angola in 2022.

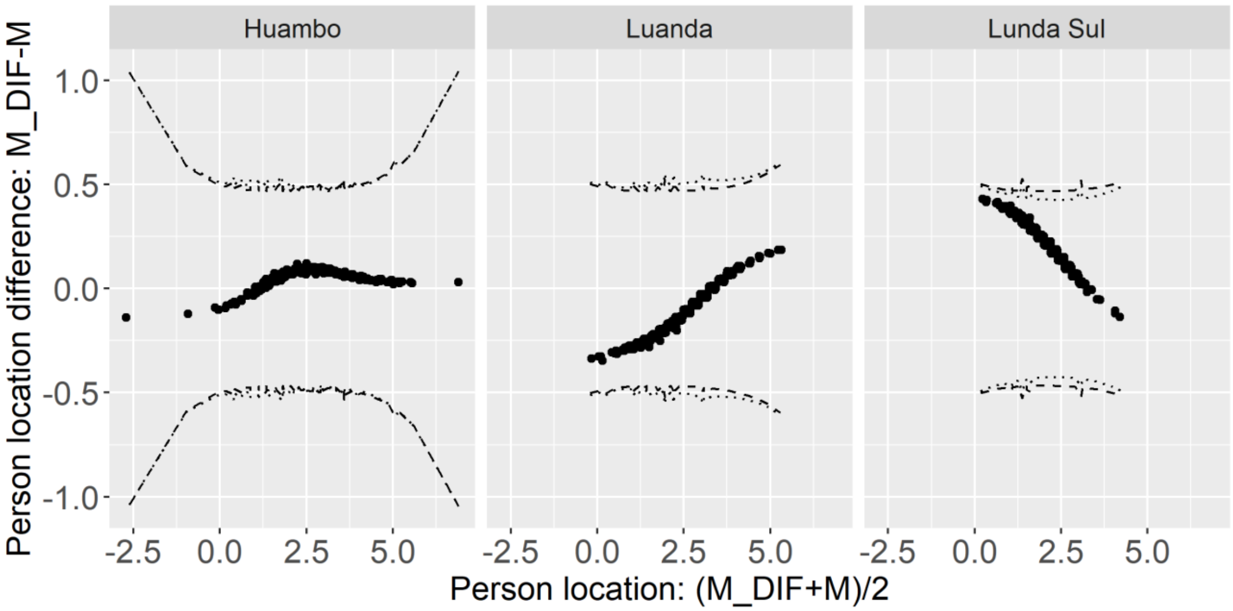

Figure I Bland-Altman plot comparing the estimated persons' locations based a model common for all three provinces (M) and on a model accounting for differential item functioning (DIF) by province (M_DIF), i.e. a model in which all items, but Freedom of expression, are province specific. The item Freedom of expression was judged as functioning the same across the provinces based on Figure G and H. The dashed(dotted) lines are the standard deviations from the estimation when ignoring (accounting for) DIF. The differences in persons' locations under the two models (y-axis) are within the uncertainty accompanying the estimation. The impact of DIF is more pronounced in Luanda and Lunda Sul, increasing the spread of persons' locations in the former and decreasing the spread in the latter case. Data the cross-sectional study with young women in Angola in 2022.

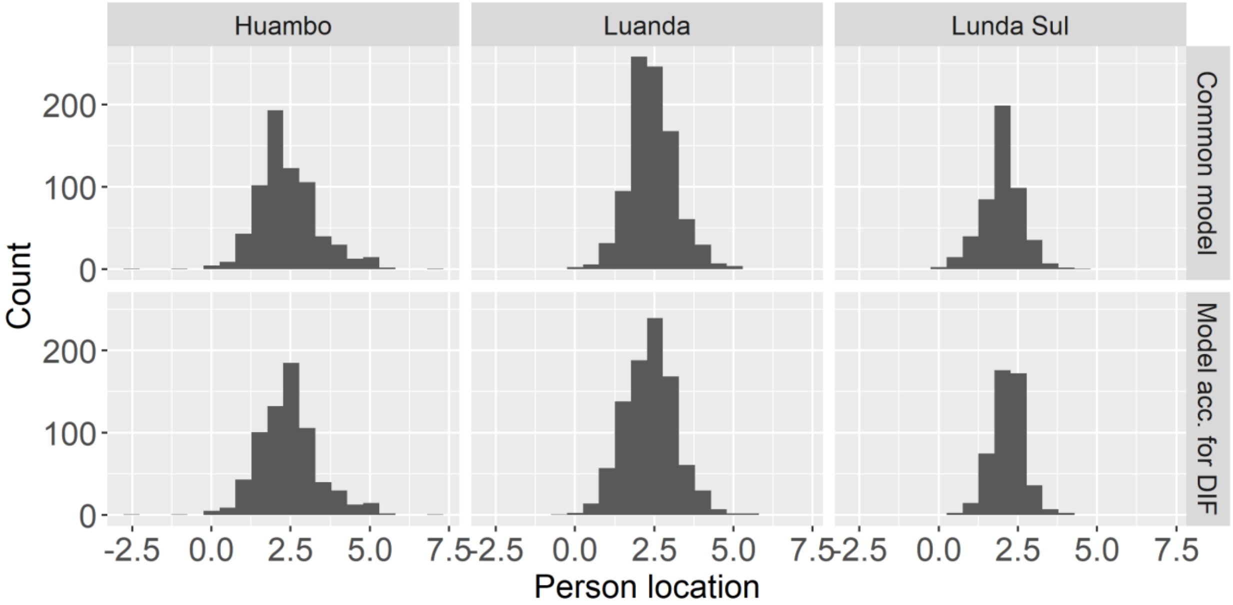

Figure J Histograms of persons' locations in the three different provinces when ignoring differential item functioning (DIF) (upper row) and when accounting for DIF (lower row). When accounting for DIF the spread of the values increases in Luanda and decreases in Lunda Sul. Data from the cross-sectional study with young women in Angola in 2022.

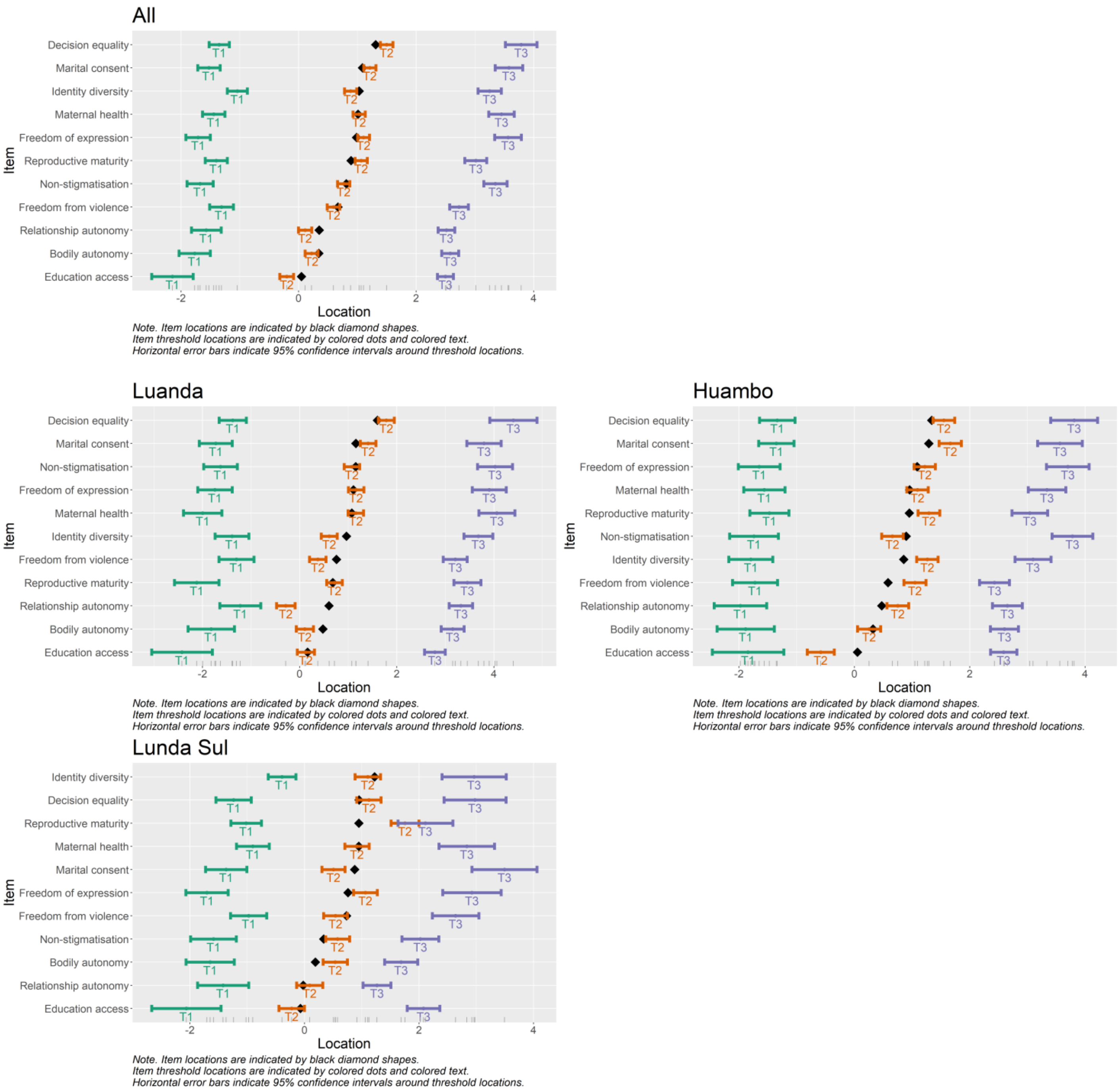

Figure K Item specific thresholds (with 95% confidence intervals) and item locations(diamonds) as estimated in the overall and the stratified (by province) analyses. From bottom to top, the items are ordered from the "easiest" to the "most difficult" as judged by their location on the logit scale. Data from the cross-sectional study with young women in Angola in 2022.
